# “Effect of mid-root perforation and its repair on stress distribution and fracture resistance: a 3D finite element analysis and in vitro study”

**DOI:** 10.1186/s12903-024-05066-z

**Published:** 2024-11-04

**Authors:** Ghada Ihab Elwazan, Nehal Nabil Roshdy, Saied Abdelaziz

**Affiliations:** Department of Endodontics, Faculty of Dentistry, Cairo University, Cairo, 12613 Egypt

**Keywords:** Root Perforation, Portland cement, Biodentine, Finite element analysis, Calcium silicate cement

## Abstract

**Aim:**

to assess and compare the effect of mid-root perforation repair using Biodentine and Portland cement in single-rooted endodontically treated mandibular premolars in terms of stress distribution using finite element analysis (FEA) and fracture resistance test.

**Methods:**

In the FEA, an extracted human mandibular premolar tooth was scanned using cone beam computed tomography, and a 3-dimensional (3D) solid model was created. A sound tooth model (ST), an endodontically treated model (ET), an instrumented and mid-root perforated and repaired by Biodentine model (BM), and perforated and repaired by Portland cement model (PCM) were the 4 models simulated. A vertical force of 300 N on the occlusal plane was applied. Evaluation of von Mises stress distribution and maximum displacement were investigated. In the fracture resistance in vitro study, 28 extracted premolars were selected and randomized into 4 groups, (*n* = 7), (A) is the negative control intact group, (B) is the positive control of endodontically treated group, (C) is mid-root perforated and repaired by Biodentine group and (D) is mid-root perforated and repaired by Portland cement. All Teeth were instrumented except for group A, group B was obturated while groups C and D were instrumented, perforated, repaired, and obturated. All groups were restored coronally except group A. Fracture force was measured; subsequently, the fracture repairability was evaluated. Finally, the data were statistically evaluated using one-way analysis of variance (ANOVA); the significance level was set at *P* ≤ 0.05 and the repairability of teeth after fracture was correlated to the maximum loading using Pearson’s coefficient tests.

**Results:**

In FEA, Maximum von Mises stress was descending assorted as 121.1 MPa for ET, 115.6 MPa for BM and PCM, and 109 MPa for ST, and in the mid-root area or perforation site were 20 MPa for PCM, 16.17 MPa for BM, 10.16 MPa for ET and 8.1 MPa for ST while the Maximum Displacement was descending assorted as 0.0179 mm for ET, 0.0169 mm for BM and PCM and 0.0151 mm for ST. In the fracture resistance test, Group A showed higher fracture resistance than other groups significantly. There was a non-significant difference between Groups B, C, and D. There was also an insignificant correlation between the maximum loading and the repairability of the tooth after fracture.

**Conclusion:**

FEA and fracture resistance test showed that the 2 repair materials are acceptable and recommended in iatrogenic mid-root perforation.

**Supplementary Information:**

The online version contains supplementary material available at 10.1186/s12903-024-05066-z.

## Introduction

 Endodontically treated teeth are widely considered to be more susceptible to fracture than vital teeth. The most often reported reasons are the dehydration of dentin after endodontic treatment, excessive pressure during obturation, and the removal of tooth structure as a result of trauma, caries, or mechanical preparation during endodontic treatment [1]. 

Root perforations can be considered one of the most common procedural errors during root canal therapy or post-placement that is highly recommended in anterior and premolar teeth with significantly compromised tooth structure as fewer axial walls present to support the restoration Perforations have a negative impact on the prognosis [2, 3] which depend on the location, size, duration that the perforation remained open to contamination, feasibility of sealing the perforation, and the material used to seal it [4]. 

The material used for perforation repair should be biocompatible and should provide an adequate seal, resistance to dislodgment, and the masticatory forces on the tooth [5]. 

However, Biodentine, Portland cement and mineral trioxide aggregate (MTA) are materials that induce tissue regeneration. Portland cement was selected as one of the repair materials as it showed similarity to MTA macroscopically, microscopically they both have adequate radiopacity and alkaline nature, which demonstrated successful sealing ability [6], biocompatibility, low solubility, the capability to induce odontoblastic differentiation and when evaluated in X-ray diffraction analysis, it also supported matrix formation in a similar way in osteoblast-like cell cultures and the apposition of reparative dentin [7], it supports the role of calcite crystals and fibronectin as an initiating step in the formation of a hard tissue barrier [8], and finally lesser cost. Conversely, they may cause coronal staining, as well as problems attributed to their prolonged setting time and handling [9]. 

On the other hand, Biodentine (Septodont, Saint-Maur-des-Fossés, France) is a recent calcium silicate-based and advertised as a ‘bioactive dentine substitute’ and introduced as an alternative to MTA. Many studies approved and recommended its use as it is easy to manipulate, has a short setting time of approximately 12 min, and has a high alkaline pH [10]. It is claimed to possess better physical properties such as increased compressive strength, push-out bond strength, density, porosity, colour stability, and better biological properties such as the immediate formation of calcium hydroxide, higher release, and depth of incorporation of calcium ions [11, 12]. 

Even if the perforation area is repaired with a biomaterial, the biomechanical response of the tooth is probably altered due to loss of tooth structure [13], and it is extremely difficult to investigate such an error and influence in vivo; therefore, finite element analysis (FEA) is an alternative method combined with a fracture resistance test to verify its results.

FEA has become a powerful technique in dental biomechanics because it allows the calculation of stress, strains, and deformations in a discretionally shaped three-dimensional (3D) finite element model, and can be considered the most comprehensive method currently available to calculate the complex conditions of stress distribution as are encountered in dental systems [14]. 

FEA is viewed as a less time-consuming process than experimental research; therefore, it could minimise laboratory testing requirements and provide faster solutions. Also, it provides qualitative and comparative study methods for various test problems. Besides that, FEA can take different and repeatable experiments on a single or few typical subjects and bring in variables that cannot be approached by other means [15]. 

This study aimed to assess and compare the effect of mid-root perforation repair using Biodentine and Portland cement in single-rooted endodontically treated mandibular premolars in terms of stress distribution using the FEA and fracture resistance test. The null hypothesis has adopted the idea that there would be no difference in the stress distribution or displacement or fracture resistance in endodontically treated single teeth and teeth with mid-root perforation repaired either by Biodentine or Portland cement and intact teeth.

## Materials and methods

### (A) finite element analysis section

#### Stereolithography (STL) file creation

After approval by the Research Ethics Committee; the first 3D geometric model was constructed from the Cone beam Computed tomography (CBCT) (Planmeca Promax Classic, Finland) with the following parameters: Voxel size = 0.075 μm, kV = 90, mA = 12 and limited field of view to an extracted intact mandibular premolar. The second 3D model was constructed from CBCT after its access and mechanical preparation via ProTaper Next (Dentsply Maillefer, Ballaigues, Switzerland). The third 3D model was constructed from CBCT after mid-root perforation was performed by a diamond tapered stone with a round end from inside the accessed crown and instrumented root canal.

The three scans of the selected tooth were acquired. Importing the scans (dicom file) to Materialise mimics 20 program (Materialise Mimics; version 10.01; Materialise, Leuven, Belgium) for segmentation to acquire STL files finally refinement and smoothening were done automatically and manually by 3 Matic Medical B.O. (Materialise, Leuven, Belgium) and Autodesk Meshmixer programs (Autodesk, Inc., Mill Valley, CA, USA).

#### Development of finite element models

**Fig. 1:**
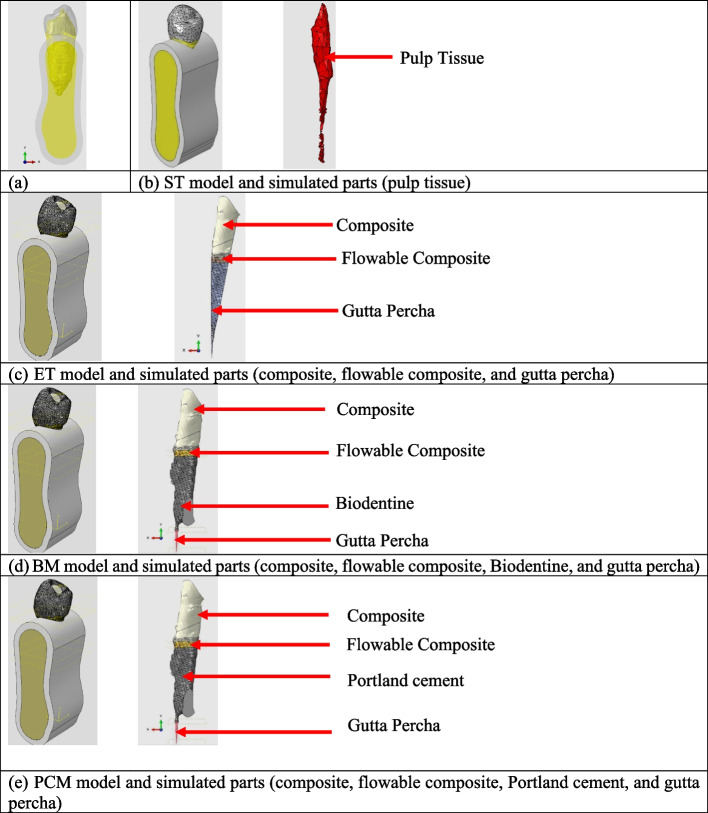
**a** Cross-sectional view for the model assembled in the jaw, **b** ST sound tooth model and its simulated parts (pulp tissue), **c** ET endodontically treated model and its simulated parts (composite, flowable composite, and gutta percha), **d** BM model and its simulated parts (composite, flowable composite, Biodentine, and gutta percha), **e** PCM and its simulated parts (composite, flowable composite, Portland cement and gutta percha)

Exporting the STL file containing 3D images to Abaqus software (Abaqus 6.14; ABAQUS Inc., Providence, RI, USA), then construction of the simulated periodontal ligament (PDL) that was assumed to be 0.3 mm, alveolar bone was obtained by growing the outer surface of the tooth from 1.5 mm below the cementoenamel junction (CEJ), and the dimensions of cortical bone and cancellous Bone to imitate the anatomy of the jaw were 10.4 mm coronal bone width, 10.0 mm apical bone width, and 30.7 mm as cross-section height [16] Fig. 1.

Then categorize the models into 4 models:

- (ST) model that simulates a sound tooth. (ET) model that simulates an endodontically treated tooth (instrumented and obturated) and restored. There are two repair material models: the Biodentine model (BM) and the Portland cement model (PCM). Each model was instrumented, mid-root perforated, repaired by either material and restored.

Finally, the assembly of pulp tissue parts, construction of simulated gutta-percha as root canal filling material, Biodentine and Portland cement as perforation repair material in the root canal space (from the perforation site till the CEJ), flowable composite at the level of the CEJ of 1 mm width and backfilling by composite resin as a filling material in the accessed crown and a space in the untreated model Fig. 1.

All the dental tissues were presumed linearly elastic, homogeneous, and isotropic. The mechanical properties, including Young’s modulus (modulus of elasticity) (E) and Poisson’s ratio, were determined from the literature (Table 1).

**Table 1:** Simulated structures properties

Structure	Young’s Modulus (E, MPa)	Poisson’s Ratio	References
Enamel	41,000	0.31	[17]
Dentin	18,600	0.31	
Pulp	3	0.45	
Spongious bone	1370	0.3	
Cortical bone	13,700	0.3	
PDL	68.9	0,45	
Gutta Percha	0.69	0.45	
Biodentine	22,000	0.33	[12]
Portland cement	22,400	0.25	[18]
Flowable composite	7000	0.25	[19]
Composite resin	24,494	0.31	[20]

Meshing 10-node tetrahedron elements with quadratic displacement shape functions and three degrees of freedom per node (C3D10) were used for meshing. A mesh sensitivity analysis was performed initially, and accordingly, based on the optimisation process and convergence analysis to control the accuracy of FEA models, the element sizes were 0.5 mm for cortical and spongy bone, 0.3 mm for the pulp and enamel, and 0.2 mm for the dentin. After the convergence test, the elements used to create the ST model and the rest of the models were approximately 695,251 elements and 856,741 elements, respectively. These models were subjected to static loading of 300 N with a 4 mm stainless steel sphere parallel to the long axis of the root, directed at the central groove. Von Mises stress evaluation was carried out, and maximum Von Mises stress values (MPa) in the entire tooth and in the mid-root area or perforation site were evaluated. Furthermore, the maximum displacement magnitudes (mm) of the teeth were also calculated. Finally, numeric data were transformed into colour graphics to give a better visualisation of the models.

### (B) fracture resistance test

This study was in vitro, parallel controlled, in which fracture resistance and failure mode of four parallel groups were examined. It was held at the endodontic Dentistry Department laboratory at the Faculty of Dentistry, Cairo University, Egypt, after research ethics committee approval by ethics approval number 19—7—64. One-way analysis of variance power calculation for more than two groups was used to detect the proper sample size at a power of 80% and a two-sided significance level of 5%, and the mean and standard deviation were taken from a previous study [21]. At least 7 samples were assigned for each group; so the total sample size is 28 specimens.

Twenty-eight freshly extracted for orthodontic or diabetic purposes, intact, mature single-rooted, single-canalled mandibular premolars were collected. Teeth with severe root curvature, complex root and root canal anatomy, calcified root canals, internal resorption, and root caries were excluded.

Random allocation and sequence generation were performed; to prevent selection bias in the interventions, the allocated sequence was protected and concealed until assignment using sequentially numbered opaque sealed envelopes in which the teeth would be placed, and to prevent detection bias, the outcome assessor and the statistician were blinded.

#### Sample preparation

The collected teeth were cleaned from any hard deposits by using an ultrasonic scaler and were disinfected in 2.6% sodium hypochlorite for 2–3 h. The teeth were then stored in saline solution until use.

Group A, (*n* = 7) Control group (Intact teeth) were isolated. The rest of the teeth were accessed by round bur and Endo Z; the canals were negotiated with K-files till size #25 to establish the glide path, and then the working length was adjusted to 1 mm shorter after the first inspection of the file at the apex.

The ProTaper Next files (Dentsply Maillefer, Ballaigues, Switzerland) were used in a sequential manner of X1 (#17/0.04), X2 (#25/0.06), X3 (30/0.07) up to reach apical preparation by X4 (#40/0.06) Finishing File using an endodontic motor with adjusted torque of 4 Ncm and speed of 300 rpm according to the manufacturer’s instructions. The rotary files were introduced inside the canal using EDTA gel (MD-Chelcream, Meta Biomed Co. Ltd, Korea) and irrigation was done by sodium hypochlorite solution (Dent House, Medical company, Cairo, Egypt) of 2.5% concentration between consecutively files by side vented needle gauge 30 (ENDO-TOP. Irrigation Needles, Poland) and dryness by paper points size #40.

All the teeth were then irrigated with 5 ml of normal saline and dried by paper points size #40 (Meta Biomed Co. Ltd, Korea).

#### Grouping

Teeth were divided into 3 groups:

- Group B: Teeth endodontically treated (instrumented and obturated) group.
- Group C: Biodentine group.
- Group D: Portland Cement Group.

**Fig. 2:**
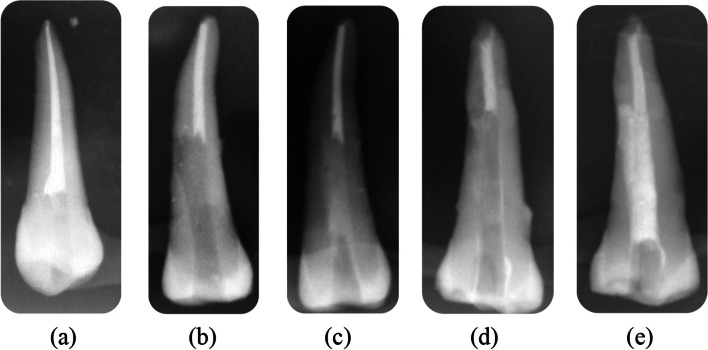
**a** Post-obturation radiograph in group (B), **b** Radiograph after apical seal and before perforation repair in group (C), **c** Post obturation radiograph in group (C) after perforation repair by Biodentine, **d** Radiograph after apical seal and before perforation repair in group (D), **e** Post obturation radiograph in group (D) after perforation repair by Portland Cement

*Group B*: after the previous procedures, master cone verification was done, and obturation was performed by mixing the resin-based root canal sealer (ADSEAL, Meta Biomed Co., Ltd, Korea) and vertical compaction technique of the gutta-percha (Meta Biomed Co. Ltd, Korea) followed by obturating the rest of the canal by Obtura (Denjoy, Changsha City, Hunan Province, China). A radiographic examination was done to verify the obturation quality Fig. 2.

*Group C*: middle root perforation was done with diamond cutting tapered with round stone (Mani, Kiyohara Industrial Park, Utsunomiya, Tochigi, Japan) from inside the accessed crown and instrumented root canal. Master cone verification was done. Obturation was done by vertical compaction of gutta-percha #40 taper 6% and resin sealer below the level of the perforation, and a radiograph was taken to ensure there was no remnant of gutta percha on the walls. Biodentine capsules (Septodont, Saint Maur des Faussés, France) were prepared by gently tapping them to loosen the powder, opening the capsule, adding 5 drops of the single-dose container of liquid, closing the capsule, and mixing the capsule on a mixing device at a speed of 4000–4200 rotations/min for 30 s, then opened and carried out with an amalgam carrier.

Application of Biodentine in the perforation area was done by pluggers of different sizes till complete repair of the perforation area and filling the rest of the root canal till the Cementoenamel Junction. Another radiograph was taken to ensure that the perforation was repaired properly. A radiographic examination was done to verify the obturation quality and perforation repair Fig. 2.

*Group D*, middle root perforation, master cone verification, and obturation were done as group C, Portland cement (ASEC Helwan cement, Egypt) was prepared by sterilisation with gamma radiation to avoid bacterial contamination and yet preserve its characteristics. Barium sulphate was added as a radiopacifying agent, which possesses a very high specific surface area, as reported by Camilleri & Gandolfi 2010 [22]; thus, achieving excellent workability and material strength. Portland cement was mixed in a 3:1 powder-distilled water ratio and was carried using a thin amalgam carrier and incrementally placed into the perforation area, then complete repair of the perforation area was by pluggers of different sizes and filling the rest of the root canal till Cementoenamel Junction. A radiographic examination was done to verify the obturation quality and perforation repair Fig. 2.

Then groups B, C, and D teeth were restored by the following procedure:

- The teeth were cleaned from excess material by finishing tapered stone (Shofu polishing kit and diamond burs, Shofu Inc, Kyoto, Japan), using universal bond adhesive (One Coat 7 universal, Coltene, Altstatten, Switzerland) on a brush for distribution on all walls, then air thinning by air water syringe and curing this layer by a light cure for 30 s, applying flowable composite (Nexcomposite flow, META BIOMED CO. LTD, Korea) in a1 mm layer for proper adaptation on the obturation material, and curing this layer by a light cure for 40 s.

Followed by incremental packing of the composite (Composite 3 m Z250 nanohybrid, 3 M ESPE, St Paul MN, USA) in the cavity preparation by using a composite applicator and curing each layer for 40 s, then finishing the composite restoration by the finishing kit.

### Fracture resistance measurement

**Fig. 3:**
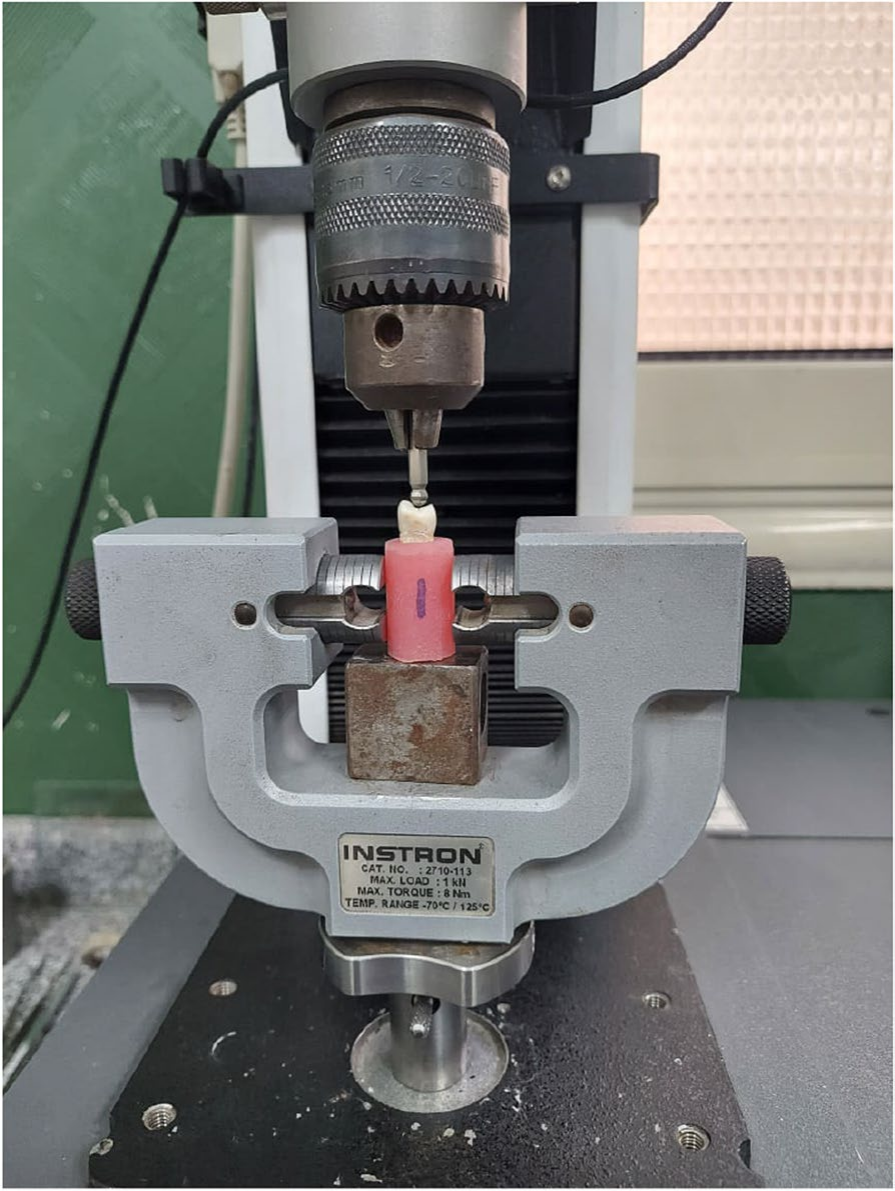
Universal Testing Machine with one of the specimens before loading

Acrylic blocks (Acrostone Co., Ltd, Cairo, Egypt) were made for all teeth in all groups in a cylindrical form of 15 mm diameter after covering their roots with wax to simulate the periodontal ligaments. Specimens were submitted to a static fracture resistance using the universal testing machine (Instron Universal testing machine, model 3345) with a 4 mm sphere stainless steel crosshead and applied to the specimens at a constant speed of 1 mm/min and parallel to the long axis of the tooth directed towards the central groove of the premolars as shown in Fig. 3. Specimens were loaded till fracture, with which maximum breaking loads were recorded in Newtons (N).

### Fracture patterns and repairability

The specimens were observed under the Stereomicroscope 70x (MA 100 NIKON, Japan) to record the pattern of each specimen, whether it is repairable as the fracture line was above the simulated bone level, while non-repairable as the fracture line was below the simulated bone level, and also vertical root fracture [23]. 

### Statistical analysis

Normality assessment was done by the Shapiro-Wilk test. Maximum load data presented in mean and standard deviation for each group with graphical representation was done. A one-way repeated ANOVA test was performed to analyse the difference between the 4 groups in fracture resistance, and the significance level was set at *P* ≤ 0.05. Data were then used for further analysis by TUKEY HSD to compare individual groups with the positive and negative control and with each other.

The repairability of teeth after fracture was correlated to the maximum loading using Pearson’s coefficient tests. Statistical analysis was performed with Excel Office 365 with real statistics add-in, Version 3.4.

## Results

### (A) finite element analysis section

 The maximum stress distribution was the greatest for the ET model of 121.1 MPa, followed by the BM and PCM of 115.6 MPa, and the least was for the ST model of 109.1 MPa. The maximum stress that reached the mid-root or the perforation area was the greatest for the PCM of 20 MPa, followed by BM of 16.17 MPa, then the ET model of 10.16 MPa, and the least was for the ST model of 8.1 MPa, as shown in Table 2.

**Fig. 4:**
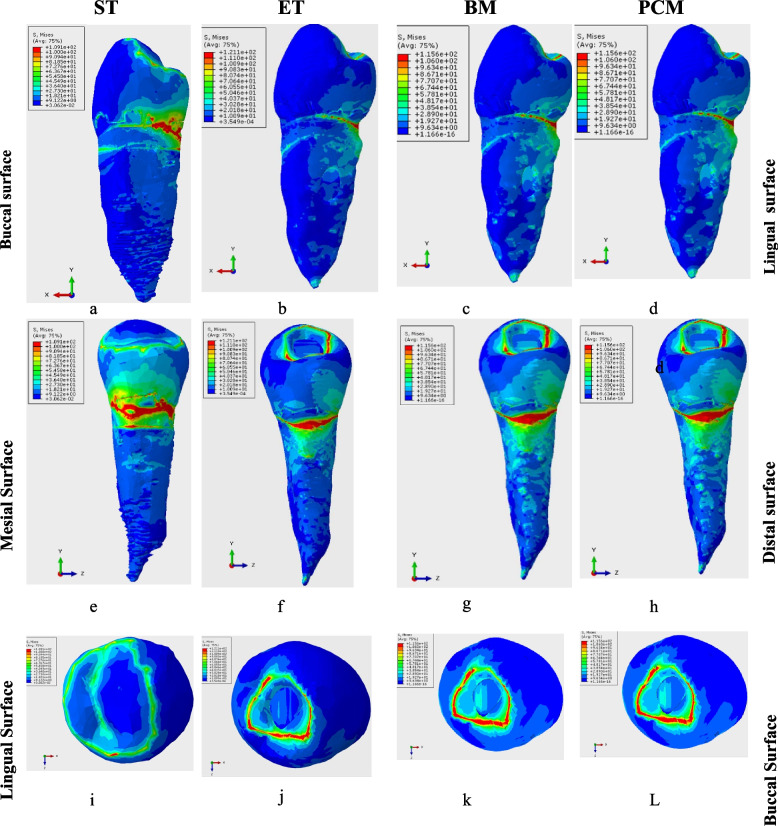
Von Mises Stress Distribution in all models; (**a**-**d**) Von Mises Stress Distribution from the Mesial aspect of the models, (**e**-**h**) Von Mises Stress Distribution from the lingual aspect of the models, (**i**-**L**) Von-Mises Stress Distribution from the occlusal of the models. ST sound tooth model, ET endodontically treated model, BM Biodentine model, PCM Portland cement model

 The von Mises stress distribution has the same pattern presentation with the greatest values at the cervical line on the lingual surface with extension to the line angles in all models from the mesiodistal and buccolingual views, but it was dissimilar from the occlusal view in which the stress distribution on the occlusal surface was on the cusps’ slopes and marginal ridges in the ST model while at the restoration margins in all other models (Fig. 4).

**Fig. 5:**
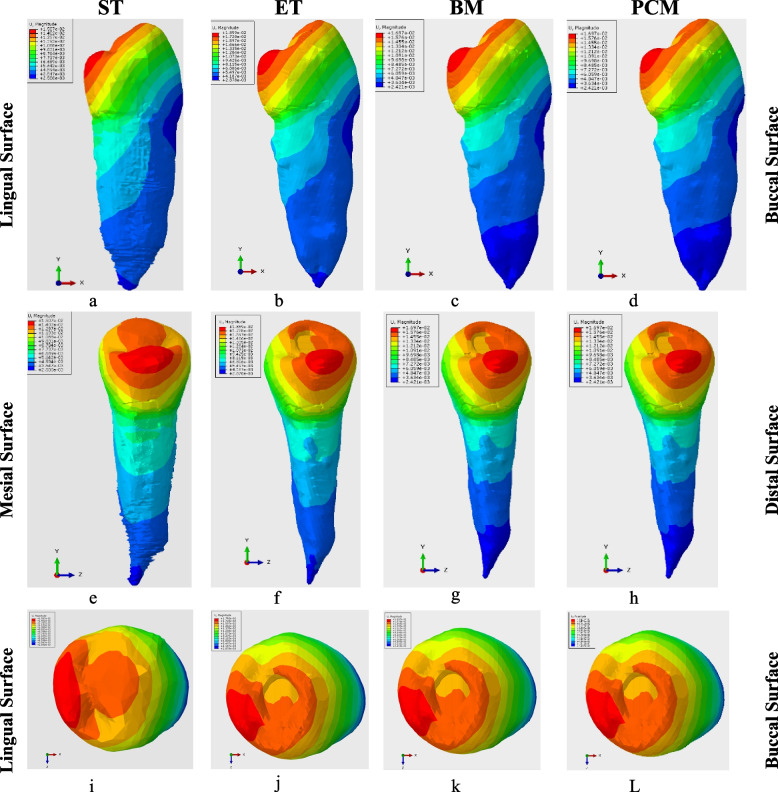
Maximum displacement in all models; (**a**-**d**) Maximum displacement from the Mesial aspect of the models, (**e**-**h**) Maximum displacement from the lingual aspect of the models, (**i**-**L**) Maximum displacement from the occlusal of the models. ST sound tooth model, ET endodontically treated model, BM Biodentine model, PCM Portland cement model

 The maximum displacement was the greatest for the ET model of 0.0179 mm, followed by the BM and PCM of 0.0169 mm, and the least was for the ST model of 0.0151 mm. These results were summarised in Table 2. The displacement values were the greatest on the cusps of the premolar, particularly the lingual cusp, as it had the maximum displacement point Fig. 5.

**Table 2:** Summary of results of finite element analysis part

Model	Mid-root or Perforation area Stress (MPa)	Overall Stress (MPa)	Maximum Displacement(mm)
ST	8.1	109	0.0151
ET	10.16	121.1	0.0179
BM	16.17	115.6	0.0169
PCM	20	115.6	0.0169

### (B) fracture resistance test

 Maximum load data is presented in mean and standard deviation for each group in Table 3. All data was normally distributed as the Shpiro wilk test was performed and yielded normal for all groups.

**Table 3:** Mean and standard deviation values of maximum load to the 4 groups

	A	B	C	D
Mean	972.8281	270.3271	301.7411	286.1603
Standard deviation	268.1396	79.75703	83.50846	87.42751

A one-way repeated ANOVA test was performed and resulted in a significant difference between all groups collectively with a p-value less than the predetermined alpha value, which is 0.05.

Data were then used for further analysis by TUKEY HSD to compare individual groups with the positive and negative control and with each other to find that a significant difference is evident when comparing any group with the negative control I.e., A-B, A-C, & A-D all show a p-value less than alpha, so a significant difference but an insignificant difference when comparing any other group with each other. However, B-C, B-D, & C-D show a p-value greater than alpha, so an insignificant difference.

**Fig. 6:**
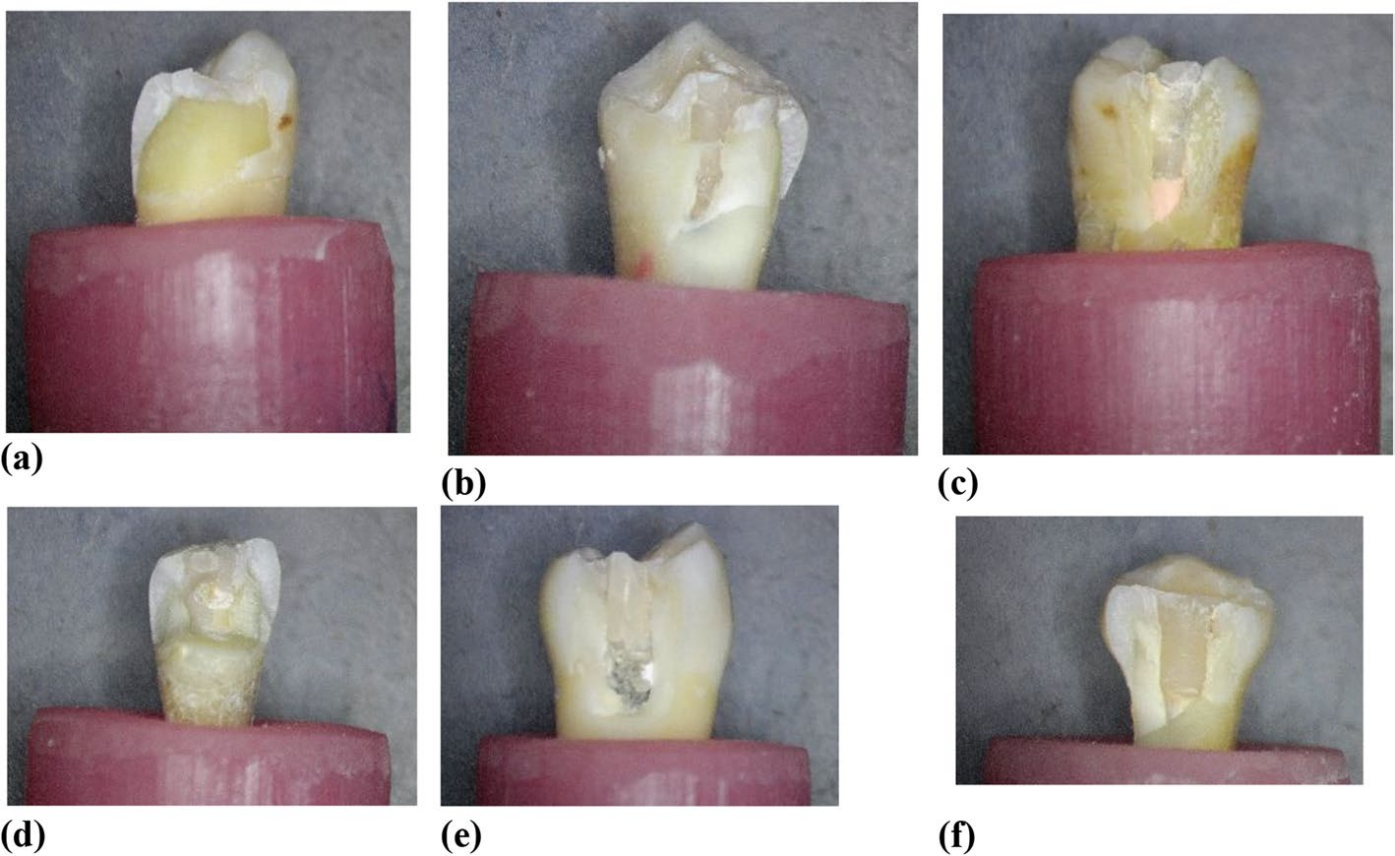
**a**-**f** Repairable fractures

**Fig. 7:**
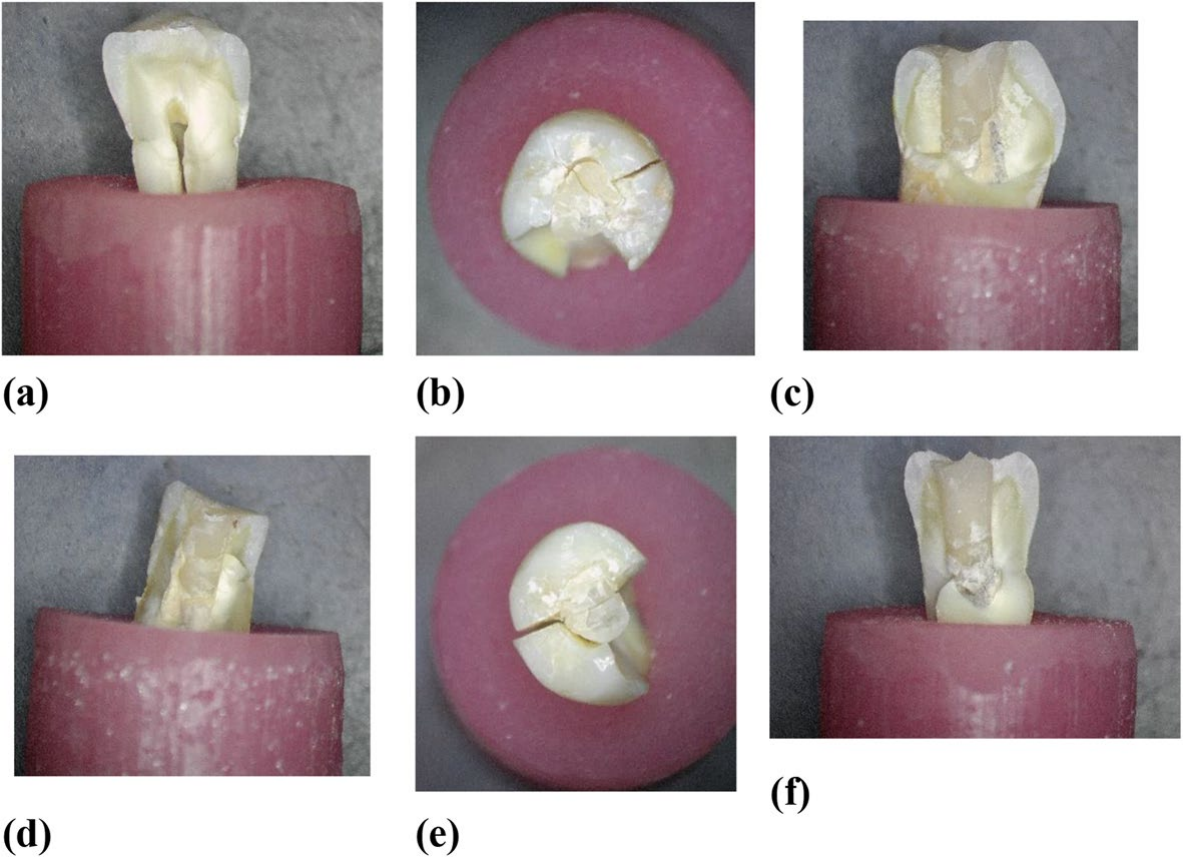
**a**-**f** Repairable fractures

 The fracture patterns were inspected and imaged; a sample of repairable fracture patterns is shown in Fig. 6, while non-repairable fracture patterns are shown in Fig. 7.

The repairability of the teeth after fracture was correlated to the maximum loading using Pearson’s coefficient tests. It revealed an extremely weak correlation with a 0.0518 correlation coefficient with a p-value of 0.8, which is more than the alpha value, which also indicates an insignificant correlation between the maximum loading and the restorability of the tooth after fracture.

## Discussion

The current study aimed to assess and compare the effect of mid-root perforation repair using Biodentine and Portland cement as examples of calcium silicate materials in single-rooted endodontically treated mandibular premolars in terms of stress distribution using finite element analysis (FEA) and fracture resistance test.

In this study, stress concentration values in root dentin were evaluated at perforation areas with non-surgical repair using Biodentine and Portland cement. For this reason, a 3D elastic FEA was performed, which allows the examination of perforation sites that are practically inaccessible in clinical conditions. We represented stress values as von Mises stresses since the von Mises stress is the combination of the numeric amount of stresses in the 3 axes (X, Y, and Z) that indicates the location of the maximum stressed areas and therefore indicates possible damage [24]. 

The highest stress values with their locations were observed in the overall tooth structure, as they are clinically important because they can reflect where cracks initiate and propagate, resulting in tooth fracture [25]. Furthermore, the maximum displacement values and corresponding areas were obtained to measure the extent of cuspal flexing results in the crack propagation in the tooth, especially the restoration/tooth interface [26]. 

Since mandibular premolars are subjected to vertical forces more than lateral forces, therefore the FEA and fracture resistance test were performed by an axial load with a steel ball directed towards the central fossa, contacting buccal and lingual cusps evenly for equal load distribution to generate the cuspal flexure and stress that challenge the coronal integrity [27–31]. 

The calcium silicates were the repair materials of choice as they induce bone and cementum formation, they can acquire their optimal strength and produce excellent sealing ability in the presence of moisture, they are very biocompatible, rarely eliciting any response from the periradicular tissues, and a cementum-like material has been consistently shown to grow directly on the material after placement with a high degree of clinically favourable long-term outcomes [7, 10, 32]. 

The overall maximum von Mises stress on the tooth structure with the occlusal loading was evaluated, the highest interpretation of stress was for the ET model with 121.1 MPa, then 115.6 MPa in BM and PCM, and the lowest von Mises stress was interpreted as 109 MPa in ST. The unexpectedly greater stress value of the ET model over BM and PCM models can be interpreted by the support of the repair materials allowing more homogenous force transfer through the contact surface between the material and the dentine after load application; consequently, decreasing the stress overall, especially at the perforation regions. Thus, filling the root canal with an adhesive material having a near dentin-like modulus of elasticity has been shown to increase the fracture resistance by creating a mono-block unit and improving stress distribution homogeneity, unlike the gutta percha, which has a modulus of elasticity of 0.69 MPa. The difference in modulus of elasticity between the dentin and repair materials is far less than the difference between dentin and gutta-percha. This theory was adopted, although the BM and PCM models had more dentinal loss in the process of the iatrogenic perforation execution.

The maximum von Mises stress in the overall test models was not in accordance with Askerbeyli Örs et al. (2019) results [13]. In their study, they tested furcal perforation consequences, and they reported that the repaired perforation model has the highest stress values, followed by the endodontically tested model with no perforation, but the least value was for the intact model. This might be attributed to the different location of the perforation, the different tested model as it was molar; therefore, this might have resulted in different stress behaviour.

The stress distribution recorded in the models determined that the most of the stress concentrated on the occlusal surface surrounding the point where the force was applied on the models, and also the lingual, distolingual, and mesiolingual walls concentrated cervically, decreasingly into the root till the apex. Additionally, it was observed that cavity margins closer to the force load experienced the greatest stress, and the stress spread in a symmetrical pattern from the force loading point. These findings were in accordance with Benazzi et al. (2014) who investigated the different directions and loading forces on mandibular premolars and recorded the same findings with the vertical loading in the central groove of the occlusal Table [33]. Additionally, our ST model exhibited forces dissipation to the marginal ridges, which was in accordance with Jiang et al. (2018). Therefore, clinicians are advised to protect the marginal ridges if possible, as they are extremely important in increasing the fracture strength after endodontic treatment [34]. 

The highest stress distribution and concentration occur in the crown, decreasingly towards the root till the apex in all models. In the ST model; this pattern was seen as it was intact with a homogenous uninterrupted structure with no introduced point of weakness, so smoother, more homogenous stress distribution occurred, while this pattern was seen in the ET, BM, and PCM, owing to the presence of composite restoration that has reinforced the crown and allowed less stress propagation to the root dentin [27, 34–36]. 

Additionally, mid-root von Mises stress was observed, the highest value was 20 MPa in PCM, then 16.17 MPa in BM in the repaired perforation sites, then 10.16 MPa in ET, and the lowest von Mises stress was observed in ST for 8.1 MPa. As an explanation to these findings, the intact tooth model presented the least stress value because of enamel and dentin preservation, continuity, and full mechanical functionality that increased the resistance to stress. The ET model showed a low von Mises stress value, which is extremely close to the ST model and can be attributed to the conservative endodontic treatment performance with no iatrogenic errors that could lead to additional loss of dentine structure.

On the other hand, the stress values of BM and PCM were different in the repaired perforation sites, although both are calcium silicates; this could be ascribed to their different modulus of elasticity values, as the Biodentine used in BM had 22,000 MPa while the Portland cement in PCM had 22,400 MPa. When comparing these values to the dentine modulus of elasticity that had 18,600 MPa, we will find that Biodentine’s modulus of elasticity is closer to dentin than Portland cement, so the modulus of elasticity of the repair material appears to affect the stress distribution in perforation sites. Both BM and PCM von Mises stress values in the repaired perforation sites were higher than the ET model and ST model because eventually there was a loss of dentine structure in the root because of the iatrogenic perforation that led to impaired structural integrity, therefore more stress concentration occurrence than non-perforated models.

The maximum von Mises stress results in the perforation area in our study were in accordance with Aslan et al. (2021) [37] in the Strip perforation test models, whom reported that the 2 repair materials were close to each other in values and exhibited higher values compared to the sound intact model but were in disagreement in their post-drill perforation test models which are much closer in design to our test models than the strip perforation test models; they recorded higher stress values in their sound tooth model than the 2 repair materials models, this might be attributed to the different force application which was an oblique force resulting in moment effect that became stronger and increasing the stress when moving from the occlusal area to the apical region, moreover, high stress in the surrounding dentin tissue, which was located closer to the apical was recorded, they added additional theory concerning the repair materials absorbing most of the stress therefore, they decreased the stress values.

For the maximum displacement of the tooth structure with the occlusal loading, results were recorded as follows: the greatest displacement was for the ET model, which was 0.0179 mm, then 0.0169 mm in BM and PCM, and the least displacement was recorded in ST for 0.0157 mm. This displacement is considered cuspal flexure, which is an increase in the intercuspal distance, so the less the effect, the more resistance there is. Heymann et al. (1991) reported that mandibular teeth undergo greater tooth flexure due to the lingual inclination of clinical crowns and smaller cross-sectional dimensions in the cervical area [26]. Moreover, both the amount of tooth tissue lost and the location of the loss were found to decrease tooth stiffness [38], Even a small reduction in tooth structure has been reported to produce a significant decrease in tooth rigidity, and deeper cavity preparations have also been related to a higher cuspal deflection [39, 40]. This explains our finding, as the lowest displacement was in the ST model in comparison to other models. The higher displacement of the ET model over the BM and PCM, although the BM and PCM had more dentinal loss than the ET model, can only be anticipated by the close modulus of elasticity of the repair materials to the dentin, and the chemical bonding with the contacting dentinal walls resulted in more reinforcement generation and support for the overlying composite restoration. Eventually the tooth, on the other hand, the gutta-percha has a very low modulus of elasticity that neither is near to the dentin nor can support the overlying composite restoration.

Our maximum displacement magnitude results were in accordance with Askerbeyli Örs et al. 2019 results [13], as they reported that the magnitude was the highest in the endodontically treated model with no perforation, followed by the repaired perforation model, while the intact model has the least value.

In the in vitro part of our study, the fracture resistance test was performed on our 4 groups, A (intact teeth), B (endodontically treated teeth), C (perforated and repaired by Biodentine), and D (perforated and repaired by Portland cement). The results showed a statistically significant difference between group A and any other group, proving the intact teeth have the greatest fracture resistance compared to other groups, proving that any loss in the tooth structure affects the structural integrity and causes less resistance to crack propagation and fracture. On the other hand, there was no statistically significant difference between group B and groups C and D, suggesting that the calcium silicates offer root reinforcement as the modulus of elasticity of both repair materials is close to that of the dentine, so they retain stress and provide homogenous stress distribution. Finally, there was no statistically significant difference between groups C and D, which is explained by the very close properties and moduli of elasticity of both materials.

These results are in accordance with Ulusoy and Paltun (2017) [30], as they concluded that there is no statistically significant difference between the 2 calcium silicate repair materials groups explaining that the development of a hydroxyapatite-like layer between dentin and calcium silicate cement suggests chemical bonding, while it is in disagreement with it as no statistical significance between intact and repair materials groups this can be attributed to their decoronation procedure exposing the pulp space without protection from the crown in the intact group, while the root canal space was reinforced with the calcium silicate repair materials in the other groups.

The results of the FEA study have been compatible with our in vitro fracture resistance study, as the maximum stress value in the ST model was the least among the models that reflected upon the intact group A in the in vitro part, which has a statistically significant difference compared to other groups believing that intact teeth have the highest fracture resistance. Model ET compared to BM and PCM had nearby values reflecting the non-statistically significant difference among the B, C, and D groups in the in vitro part. Finally, the same values of BM and PCM were also compatible with the non-statistically significant difference between groups C and D in the in vitro part.

The limitations of FEA should be considered. As a computer solution, it does not necessarily reveal how the stress is influenced by important variables such as materials’ properties and geometrical features. When FEA is used to simulate the tooth, the available model assumes the dentin is isotropic, linear elastic, and uniform with a tissue Young’s modulus and Poisson’s ratio. However, previous studies have shown that the hardness of dentin decreases from the surface to the dental pulp cavity, which makes Poisson’s ratio and Young’s modulus not always the same throughout the dentin. It is also reported that dentin has its anatomical shapes and structures. Thus, Young’s modulus varies according to the distance from the pulp. Simulating the true structure of the teeth to obtain an objective result is still a challenge for FEA. In addition, the complicated geometry of root canals and uncertainties about their mechanical properties make it a necessity that calculated values must be corroborated by experimental measurements at certain points. These developmental defects within the teeth are often ignored in establishing an FEA model while they may initiate the tooth fracture. Moreover, most FEA experiments assume that the force distributed on the canal surface is uniform., the gutta-percha was simulated to behave like a perfect fluid, distributing the load around the canal wall equally and uniformly. In reality, this infrequently happens. A pointed force is more likely the real situation of stress in the root canal under the force of preparation or with condensation during obturation [41]. 

As the FEA is imperfect, researchers should not expect the theoretical models to be perfect because understanding dental properties and processes is under development. The FEA studies cannot replace traditional laboratory studies. It needs laboratory validation to prove its results. So, in the current study, we conducted both FEA and laboratory studies to compare and validate the results [42–44]. 

## Conclusion

Within the limitations of this study, it was concluded that the prognosis of the iatrogenic mid-root perforation repair depends mainly on the proper selection of repair material, as calcium silicate cement and the technique of backfilling, Biodentine, and Portland cement proved to be adequate materials for mid-root perforation repair as they have compensated for the dentine loss and disruption of the structural integrity, thereby decreasing the generation of stresses and their concentration, which improved the tooth reinforcement. Moreover, they improved the fracture resistance of mid-root perforated teeth, which was nearly equivalent to the fracture resistance of non-perforated endodontically treated teeth.

As Prospective collection of biological specimens for research purposes by non-invasive means à  permanent teeth if routine patient care indicates a need for extraction^1^

## Supplementary Information

Supplementary Material 1.

Supplementary Material 2.

## Data Availability

All data generated or analysed during this study are included in this published article [and its supplementary information files].

## Abbreviations

FEA: Finite element analysis
3D: 3 Dimensional
ST: Sound Tooth Model
ET: Endodontically treated model
BM: an instrumented and mid-root perforated and repaired by Biodentine model
PMC: an instrumented and mid-root perforated and repaired by Portland cement model
N: Newton
ANOVA: Analysis of variance
MPa: Megapascals
mm: millimetre
MTA: Mineral Trioxide Aggregate
STL: Stereolithography
CBCT: Cone beam computed tomography
μm: micrometre
Kv: kilovolt
mA: milliampere
PDL: Periodontal ligament
CEJ: Cementoenamel junction
E: Young's modulus (modulus of elasticity)
min: minute

## Footnotes

1 An expedited review procedure consists of a review of research involving human subjects by the IRB chairperson or by one or more experienced reviewers designated by the chairperson from among members of the IRB in accordance with the requirements outlined in 45 CFR 46.110.
