# Supplementary material for: “Effect of mid-root perforation and its repair on stress distribution and fracture resistance: a 3D finite element analysis and in vitro study”
Source: BMC Oral Health. 2024 Nov 4;24:1340. doi: 10.1186/s12903-024-05066-z (PMC11566506; doi:10.1186/s12903-024-05066-z)
Supplement: Supplementary file 1 — Supplementary Material 1. [file 12903_2024_5066_MOESM1_ESM.pdf]

Group no.	Maximum load (N)	type of fracture repairable or non-repairable	Description of the fracture
A1	694.14789	NR	Crown/Root Fracture Subcrestal
A2	695.25171	NR	Split Tooth
A3	1428.50745	R	Enamel Fracture
A4	987.80058	R	Enamel Fracture
A5	1188.83215	R	Enamel Fracture
A6	825.00256	R	Enamel Fracture
A7	990.254401	R	Enamel/ Dentin Fracture
B1	266.89053	R	Enamel Fracture
B2	252.87943	R	Enamel Fracture
B3	332.5495	R	Enamel Fracture
B4	317.05429	NR	Crown/Root Fracture Subcrestal
B5	158.80347	R	Crown/Root Fracture Supracrestal
B6	379.7373	NR	Crown/Root Fracture Subcrestal
B7	184.37538	R	Enamel Fracture
C1	294.97302	NR	Crown/Root Fracture Subcrestal
C2	222.29395	R	Enamel Fracture
C3	193.28754	R	Crown/Root Fracture Supracrestal
C4	323.14642	NR	Split Tooth
C5	265.62964	NR	Crown/Root Fracture Subcrestal
C6	410.95615	NR	Crown/Root Fracture Subcrestal
C7	401.90094	R	Enamel Fracture
D1	317.28589	R	Enamel Fracture
D2	201.60588	R	Enamel/Dentin Fracture
D3	178.46126	R	Enamel/Dentin Fracture
D4	389.86038	R	Enamel/Dentin Fracture
D5	242.50037	NR	Crown/Root Fracture Subcrestal
D6	401.41437	NR	Crown/Root Fracture Subcrestal
D7	271.99396	R	Enamel Fracture
