# Supplementary material for: “Effect of mid-root perforation and its repair on stress distribution and fracture resistance: a 3D finite element analysis and in vitro study”
Source: BMC Oral Health. 2024 Nov 4;24:1340. doi: 10.1186/s12903-024-05066-z (PMC11566506; doi:10.1186/s12903-024-05066-z)
Supplement: Supplementary file 2 — Supplementary Material 2. [file 12903_2024_5066_MOESM2_ESM.docx]

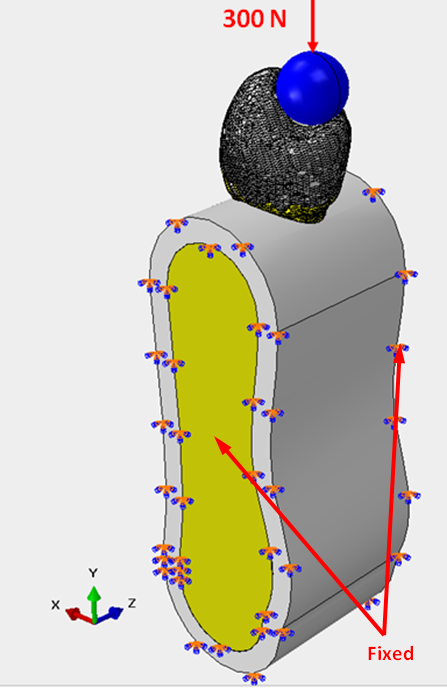


**Figure S1**

**Sound tooth model (ST)(S2-S14)**

**Assembly 3D model**


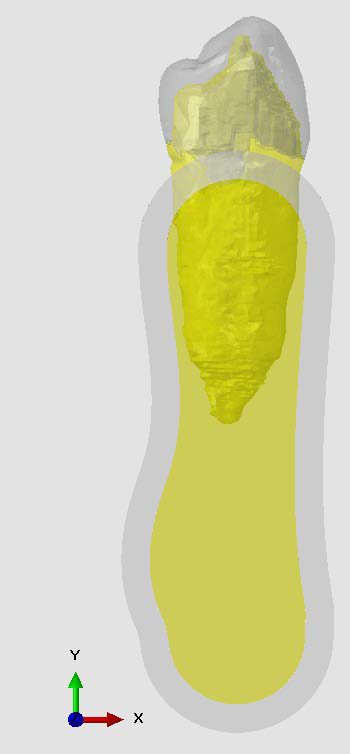

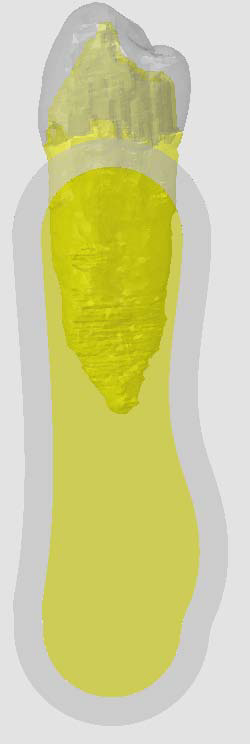


**(a) (b)**

**Figure S2: Assembly Section View (a) Distal, (b) Mesial of ST model**


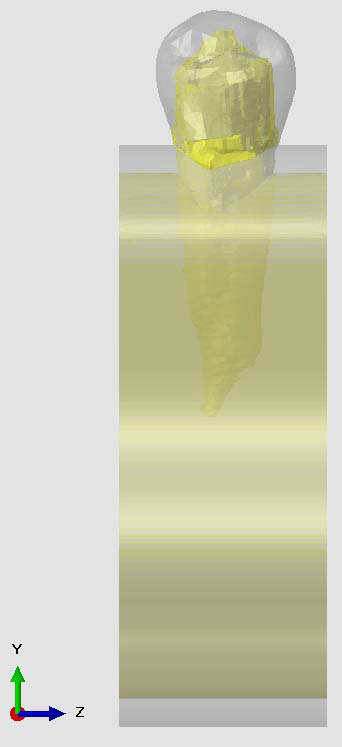

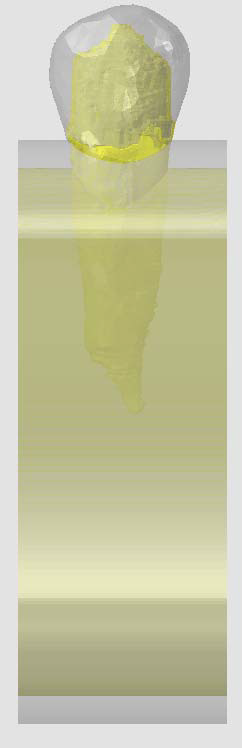


**(a) (b)**

**Figure S3: Assembly Section View (a) lingual, (b) Buccal of ST model**

**Mesh:**


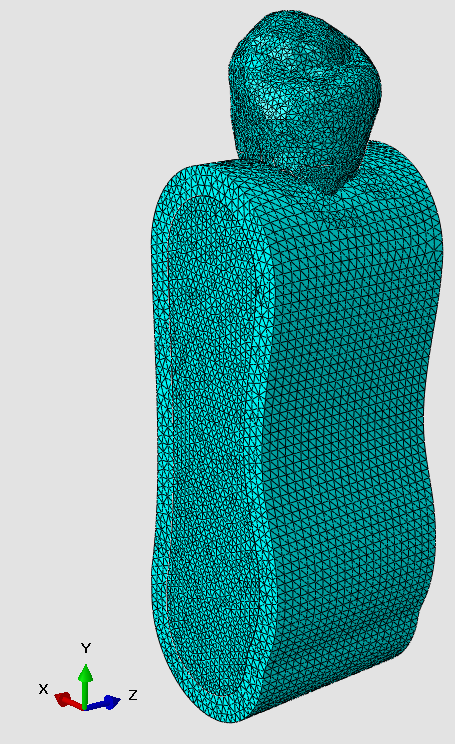


**Figure S4: Assembly Meshed 3D View** **of ST model**

**Von –Mises Stress Distribution**


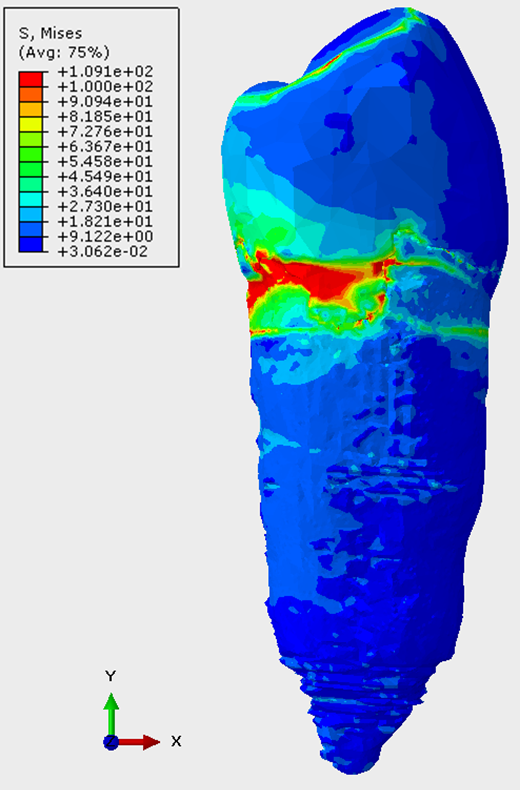


**Figure S5: Von –Mises Stress Distribution Distal View of ST model**


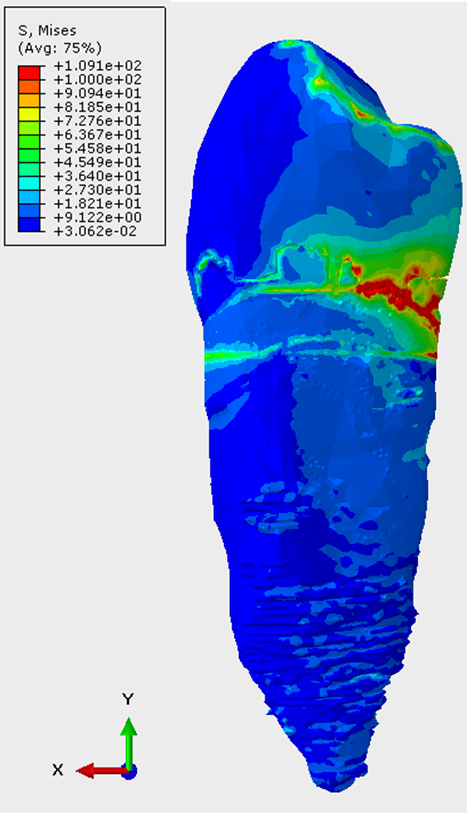


**Figure S6: Von –Mises Stress Distribution Mesial View of ST model**


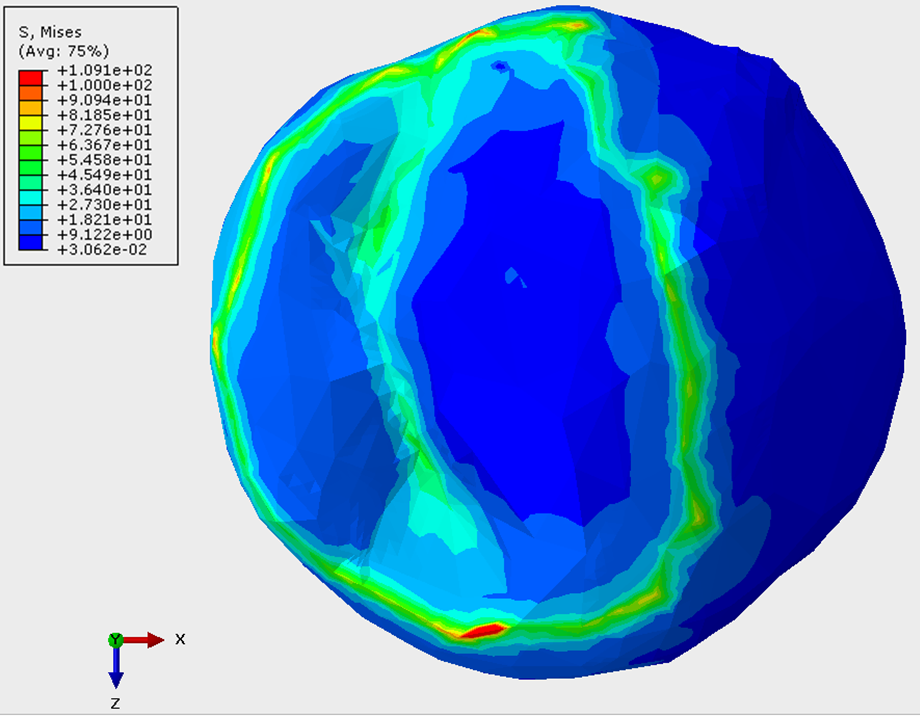


**Figure S7: Von –Mises Stress Distribution Occlusal View of ST model**


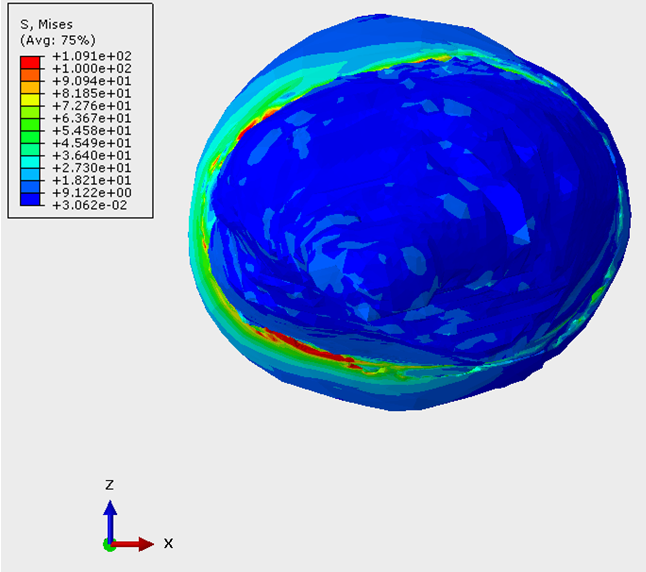


**Figure S8: Von –Mises Stress Distribution Apical View of ST model**


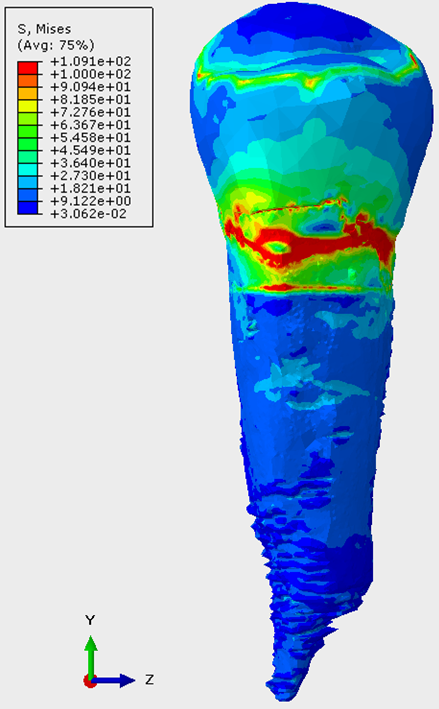


**Figure S9: Von –Mises Stress Distribution Lingual View of ST model**


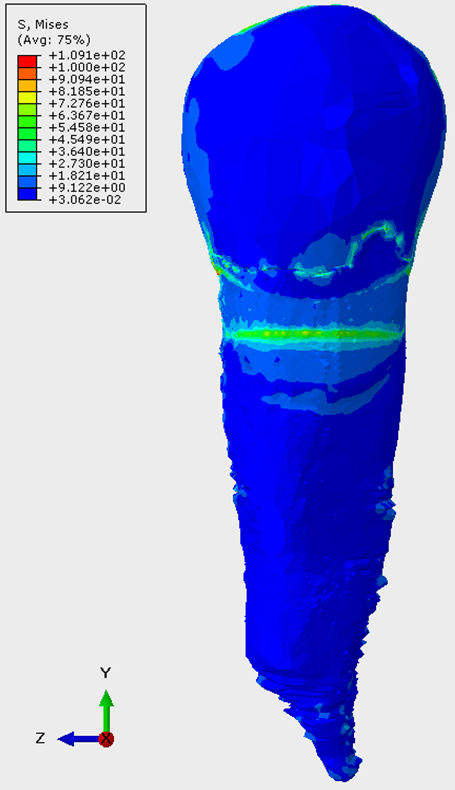


**Figure S10: Von –Mises Stress Distribution Buccal View of ST model**

**Displacement Distribution Results:**


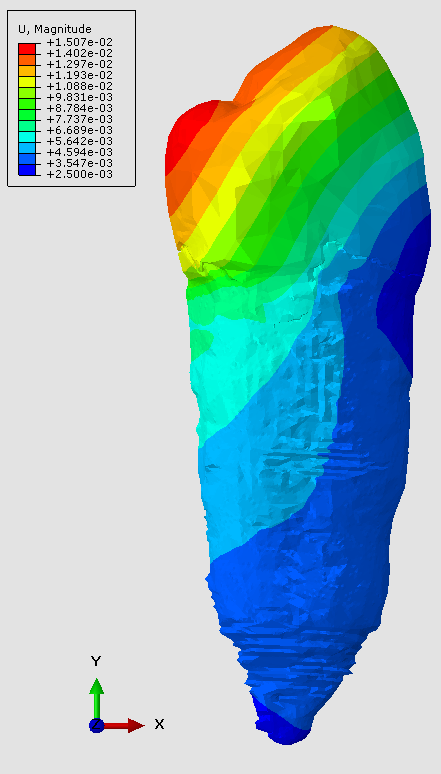

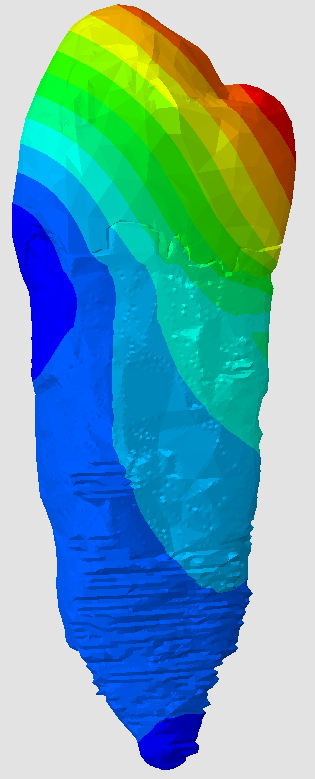


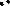

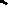

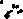

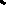


**(a) (b)**

**Figure S11: Displacement Distribution (a) Distal, (b) Mesial View of ST model**


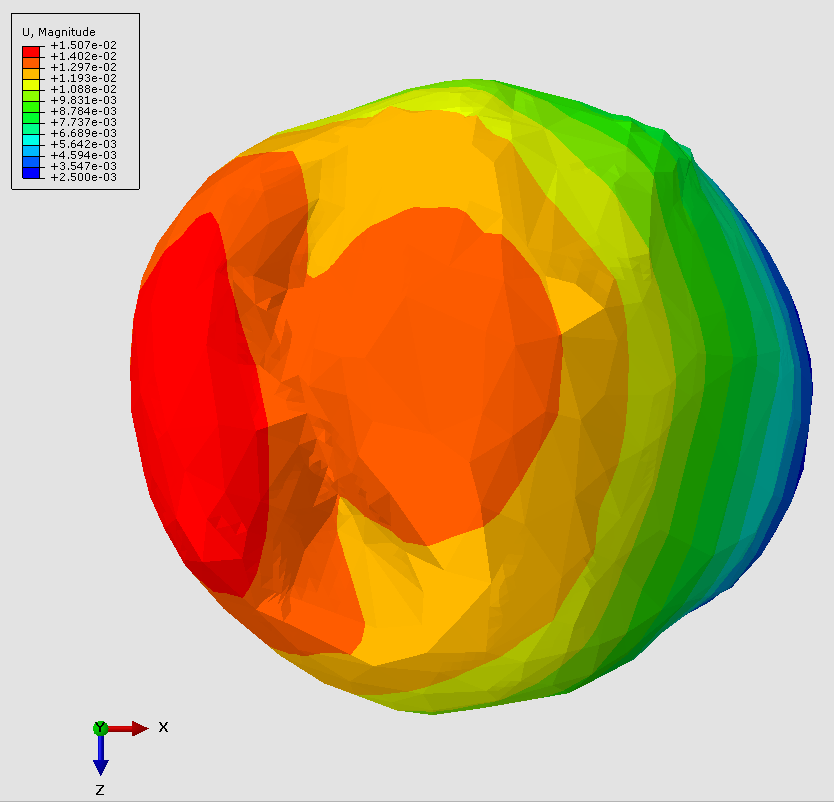


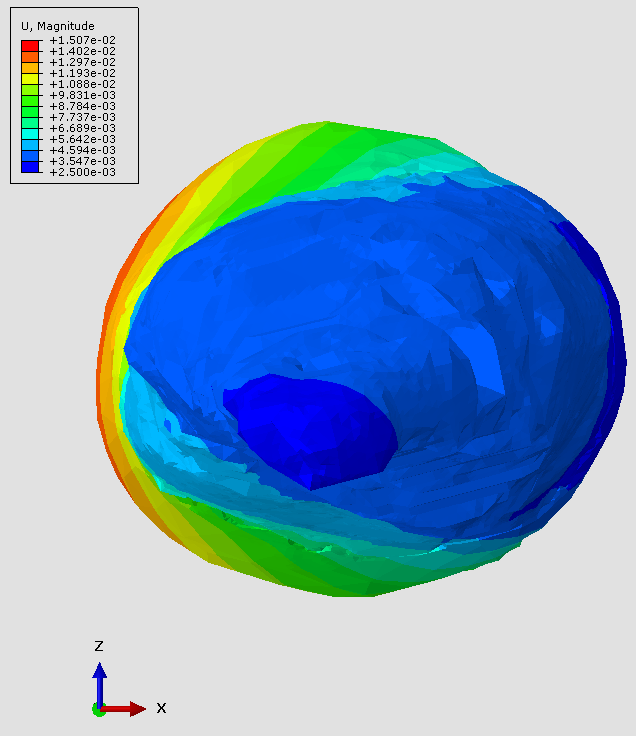
**Figure S12: Displacement Distribution Occlusal View of ST model**

**Figure S13: Displacement Distribution Apical View of ST model**


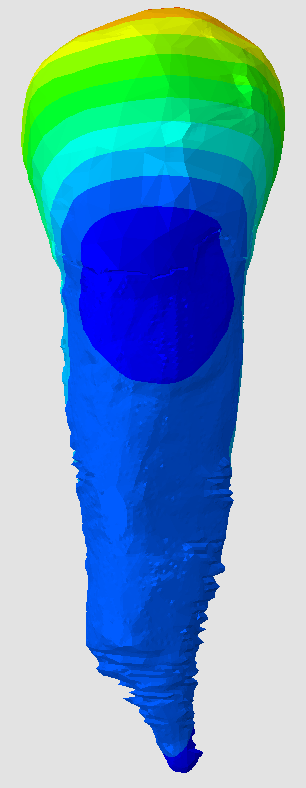

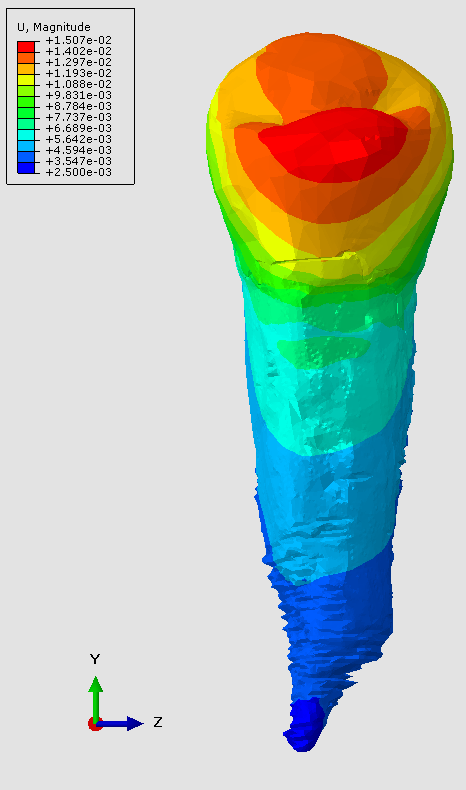


**(a) (b)**
**Figure S14: Displacement Distribution (a) Lingual, (b) Buccal View of ST model**

**Endodontically treated tooth Model (ET)(S15-S27)**

**3D Assembly view**
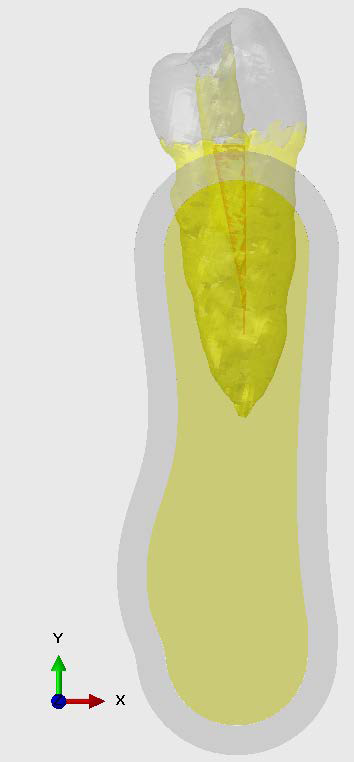

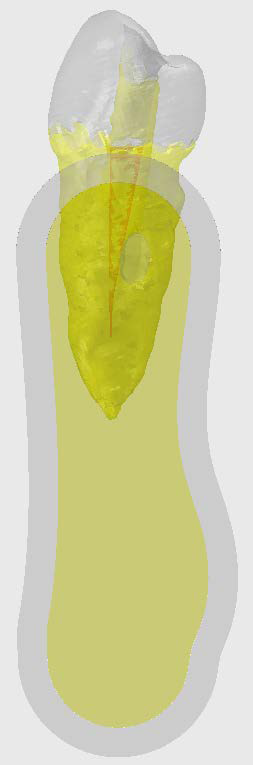


**(a) (b)**

**Figure S15:Assembly Section View (a) Distal, (b) Mesial of ET model**


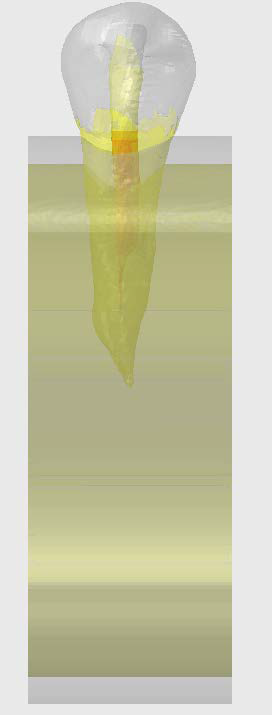


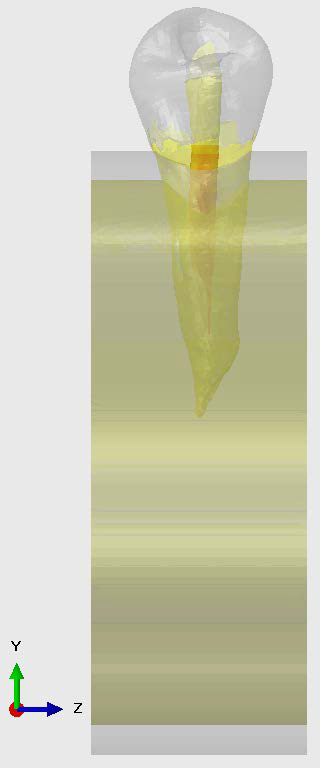


**(a) (b)**

**Figure S16: Assembly Section View (a) lingual, (b) Buccal of ET model**

**Mesh**


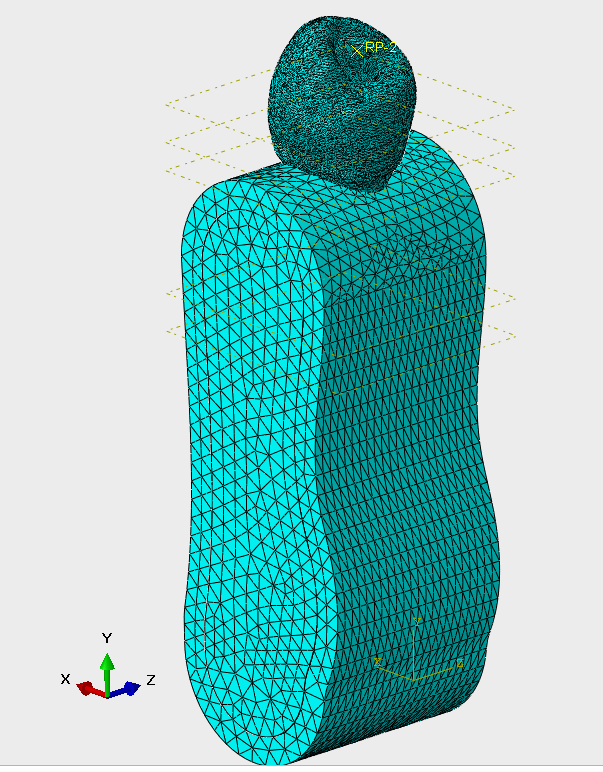


**Figure S17: Assembly Meshed 3D View** **of ET model**


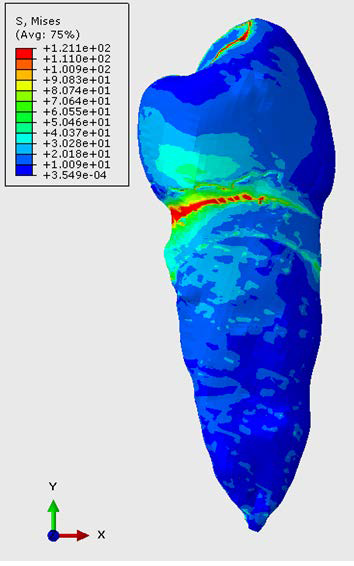
**Von –Mises Stress Distribution results**

**Figure S18: Von –Mises Stress Distribution - Distal View of ET model**


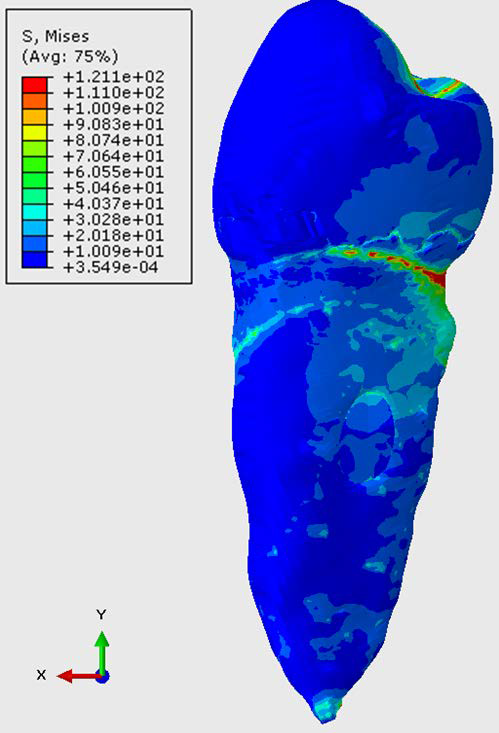


**Figure S19: Von –Mises Stress Distribution - Mesial View of ET model**


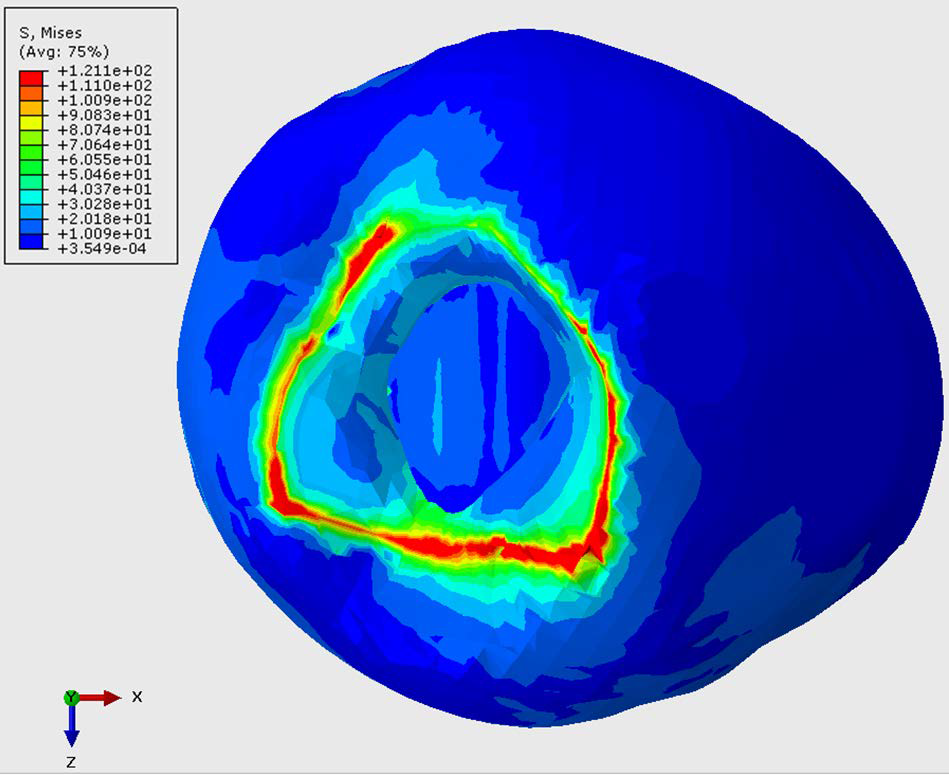


**Figure S20: Von –Mises Stress Distribution - Occlusal View of ET model**


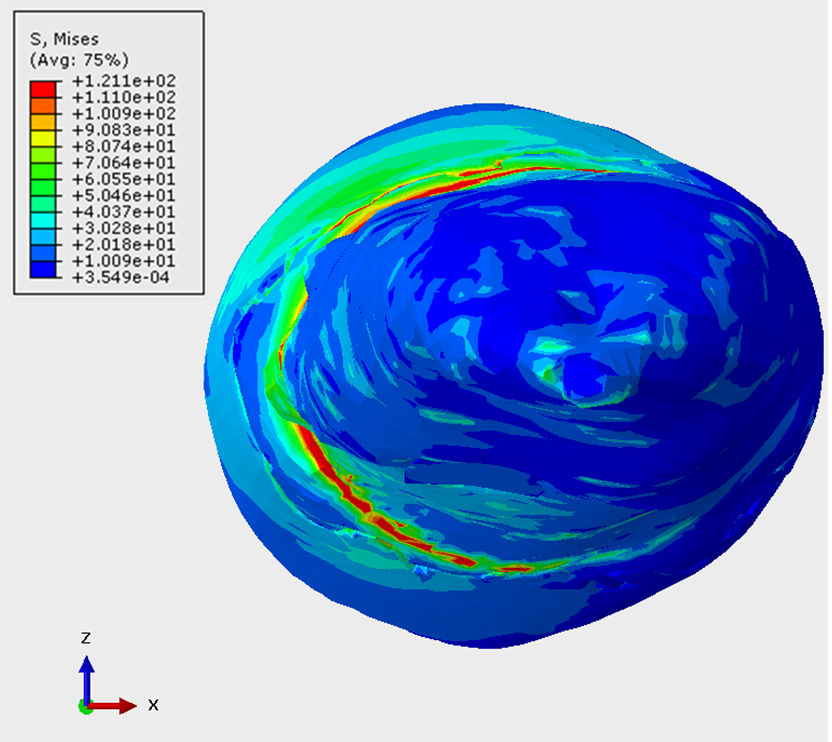


**Figure S21: Von –Mises Stress Distribution - Apical View of ET model**


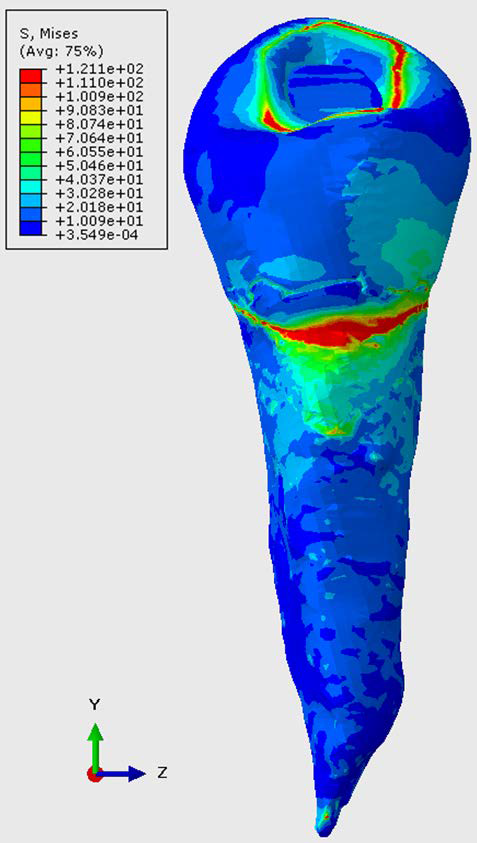


**Figure S22: Von –Mises Stress Distribution - Lingual View of ET model**


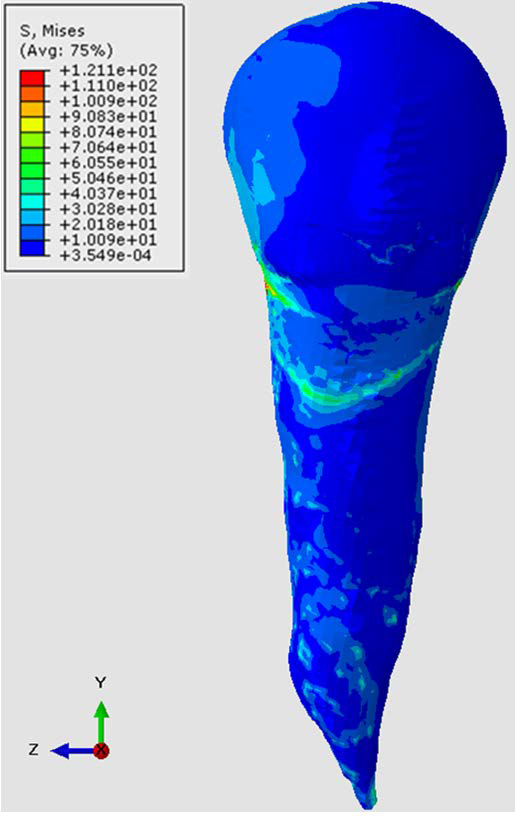


**Figure S23: Von –Mises Stress Distribution - Buccal View of ET model**


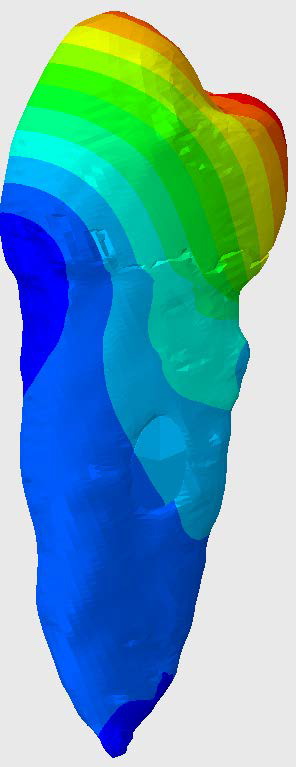

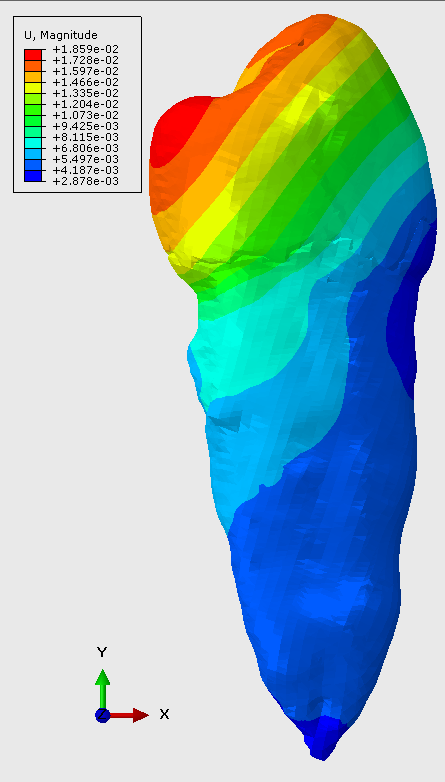
**Displacement Distribution Results**

**Figure S24: Displacement Distribution Distal/Mesial view of ET model**


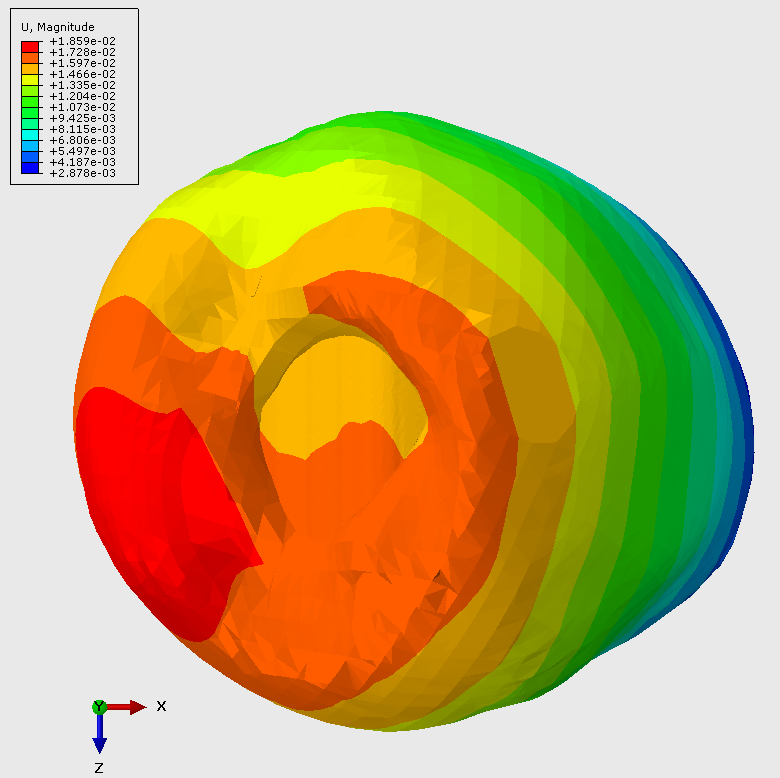


**Figure S25: Displacement Distribution Occlusal view of ET model**


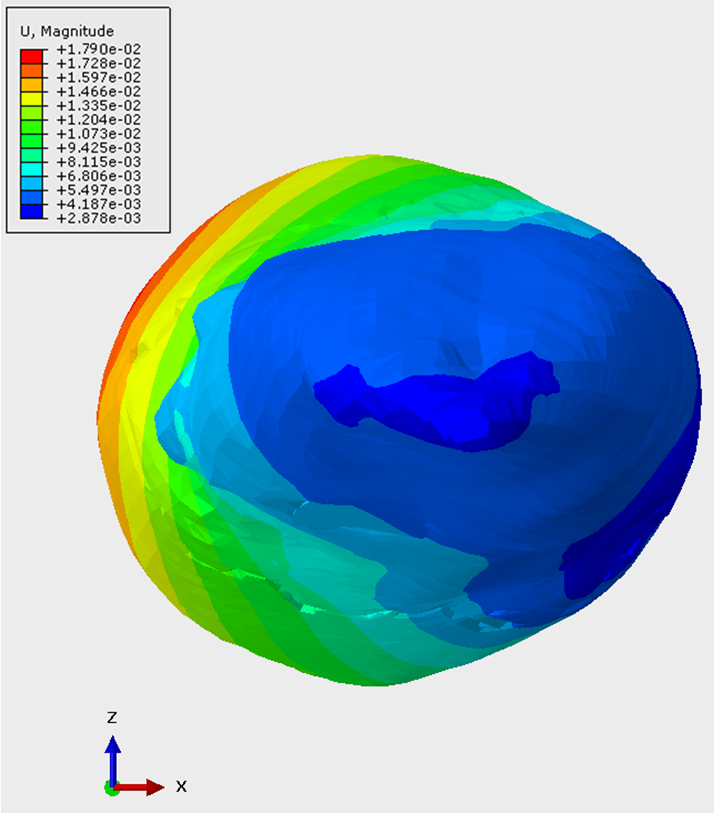


**Figure S26: Displacement Distribution Apical view of ET model**


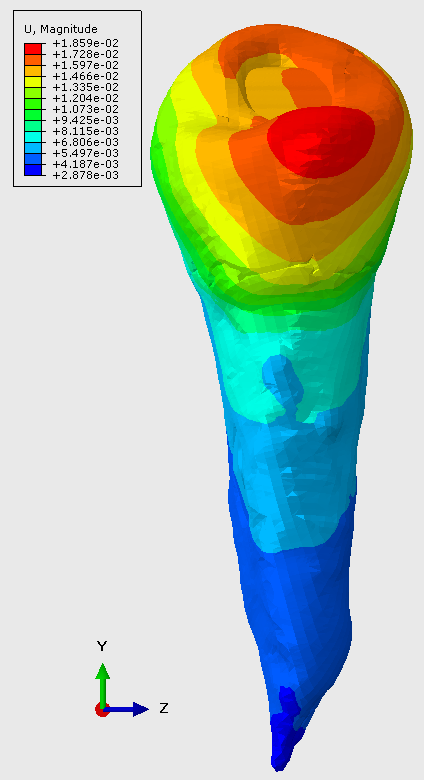

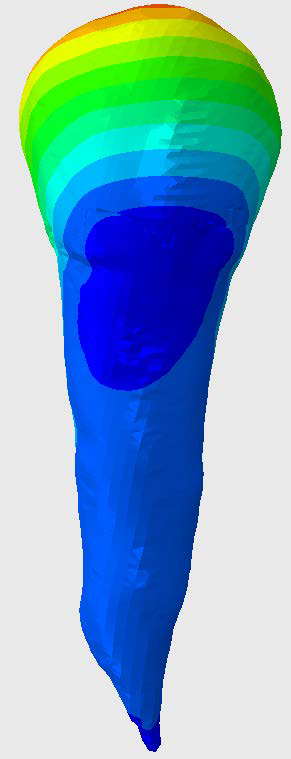


**Figure S27: Displacement Distribution Lingual/Buccal view of ET model**

**Perforated & treated by Biodentine model (BM)(S28-S41)**


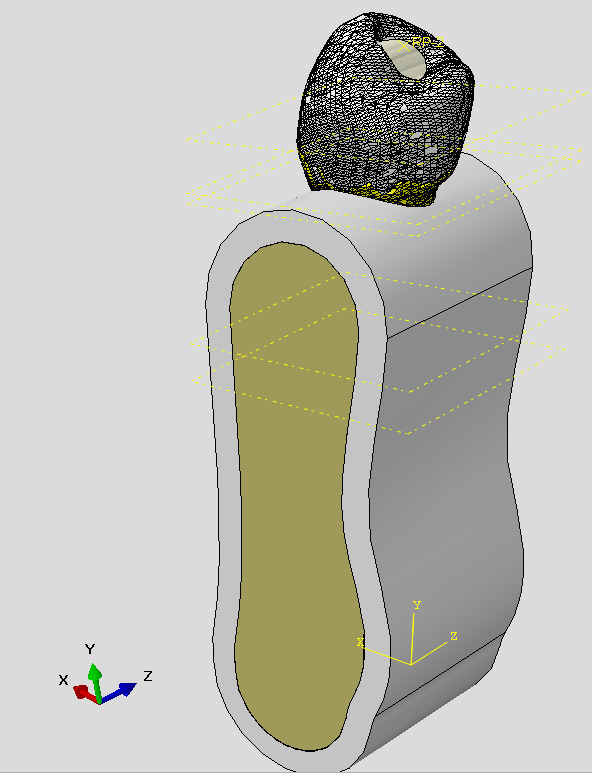


**Figure S28: Assembly 3D model of BM**


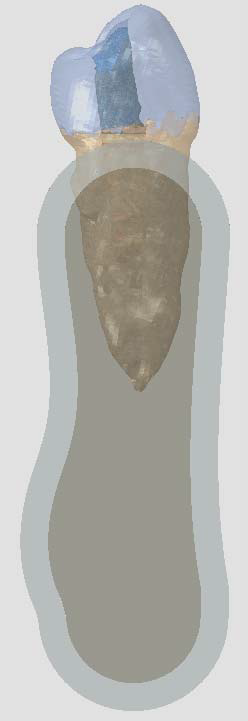

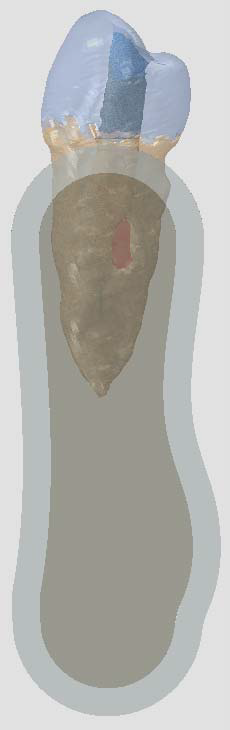


**(a) (b)**

**Figure S29: Assembly Section View (a) Distal, (b) Mesial of BM**


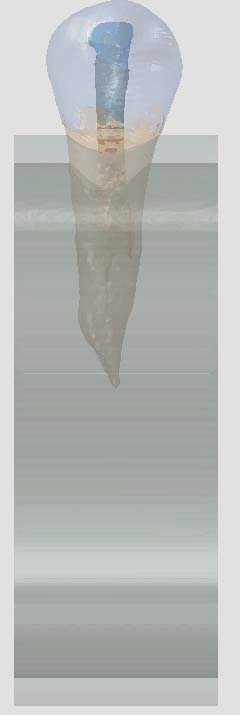

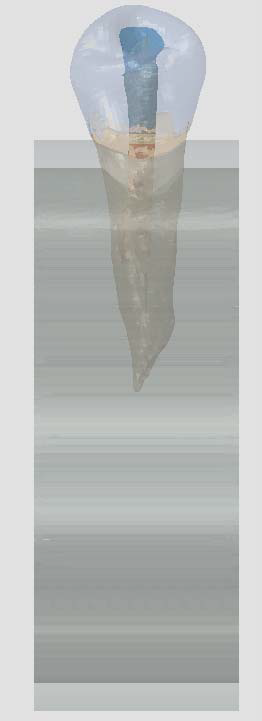


**(a) (b)**

**Figure S30: Assembly Section View (a) lingual, (b) Buccal of BM**

**Mesh**


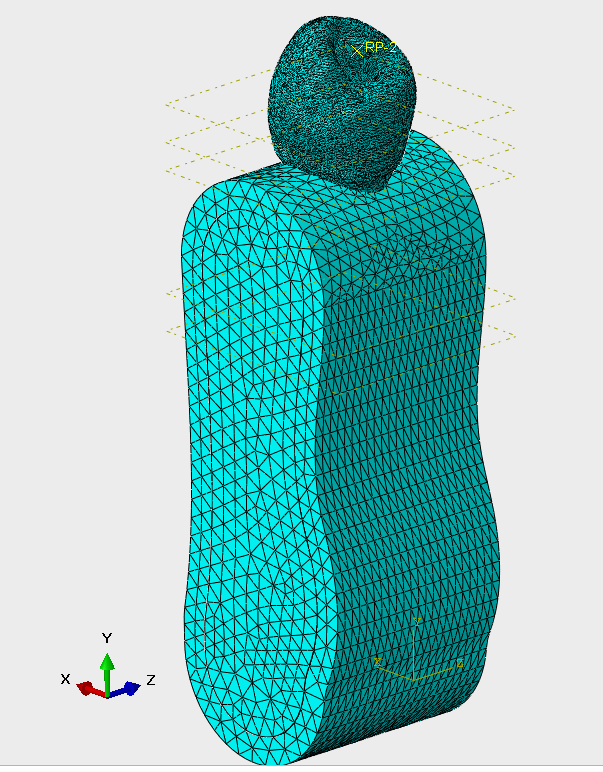


**Figure S31: Assembly Meshed 3D View** **of BM**

**Results of Scenario BM Model
Von –Mises Stress Distribution**


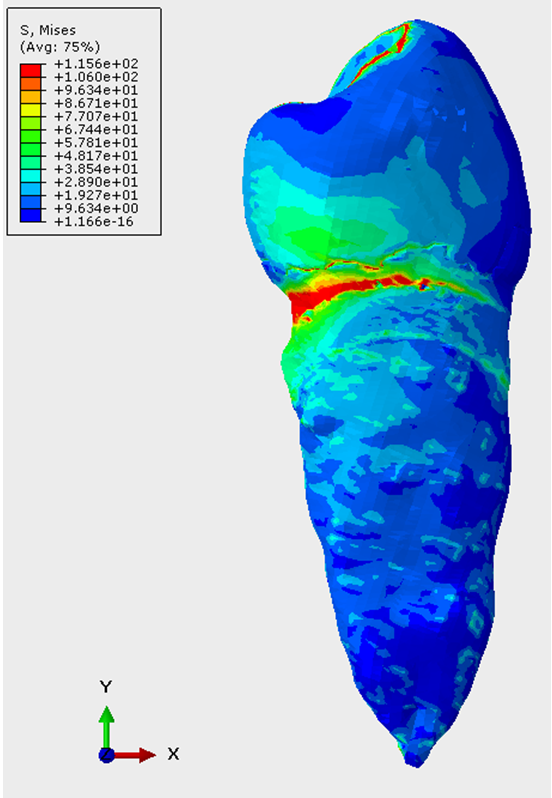


**Figure S32: Von –Mises Stress Distribution - Distal View of BM**


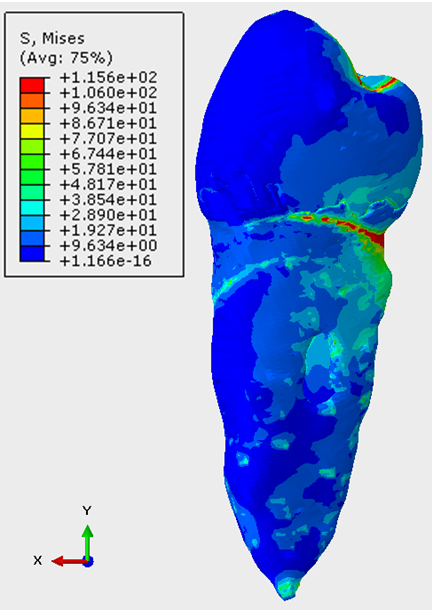


**Figure S33: Von –Mises Stress Distribution - Mesial View of BM**


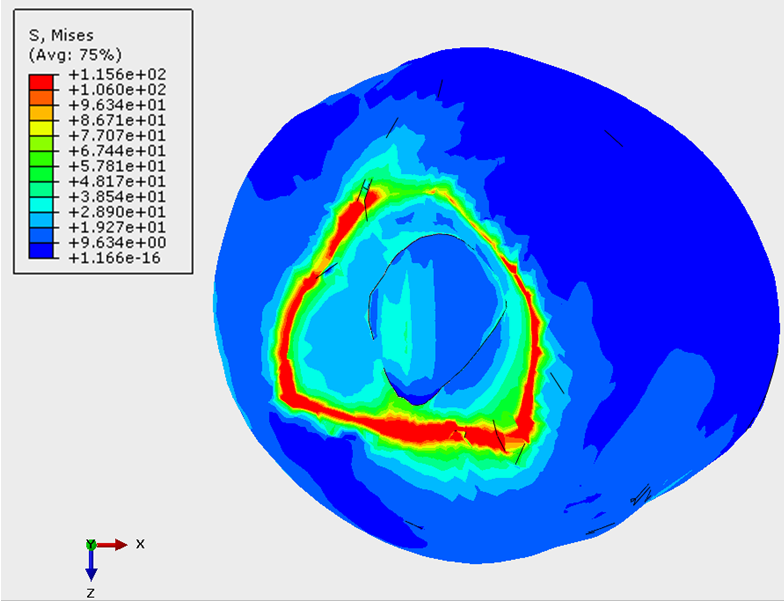


**Figure S34: Von –Mises Stress Distribution Occlusal View of BM**


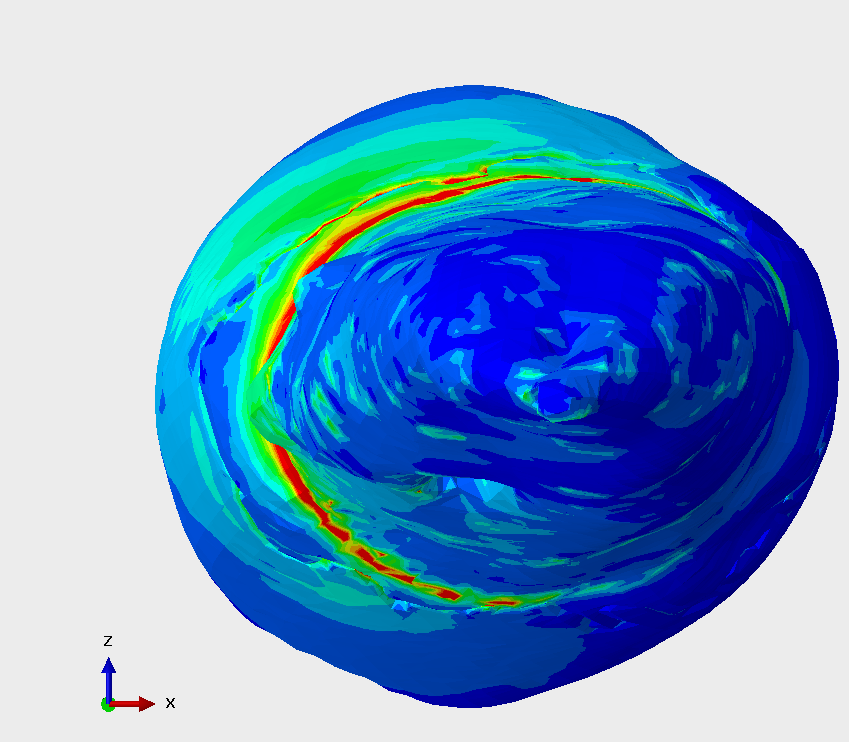


**Figure S35: Von –Mises Stress Distribution Apical View of BM**

**Figure S36:**
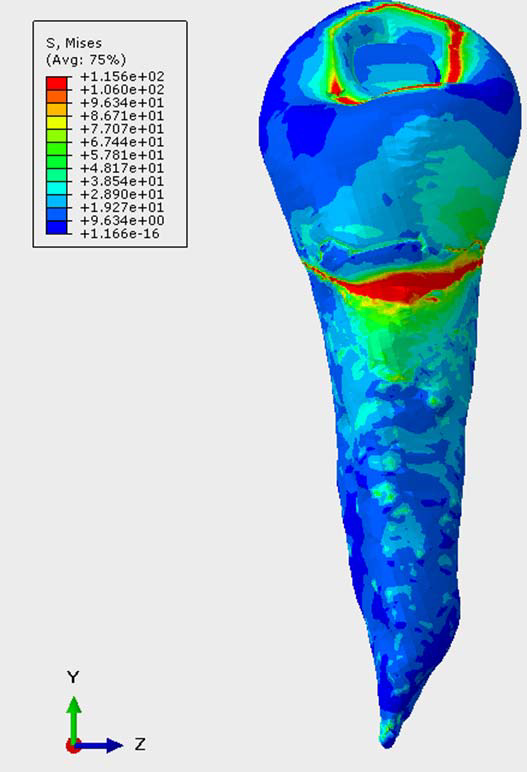
**Von –Mises Stress Distribution - Lingual View of BM**


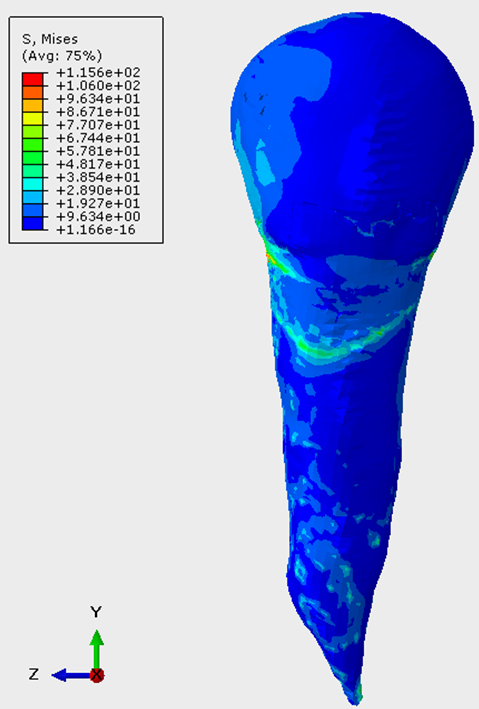


**Figure S37: Von –Mises Stress Distribution - Buccal View of BM**

**Displacement Distribution Results**


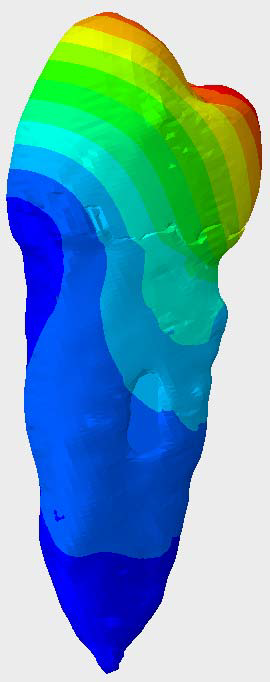

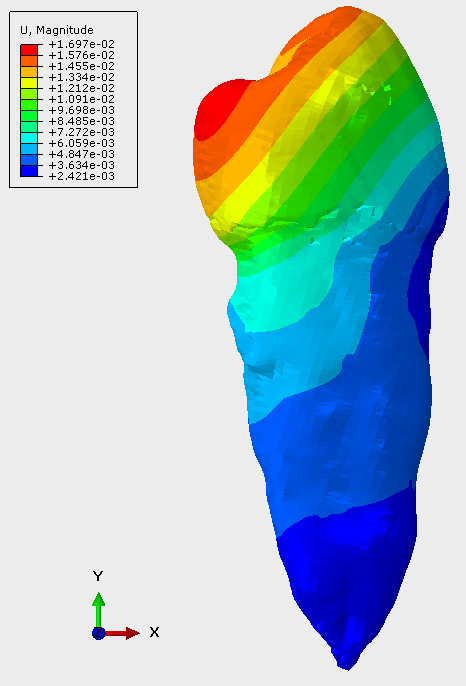


**Figure S38: Displacement Distribution Distal/Mesial view of BM**


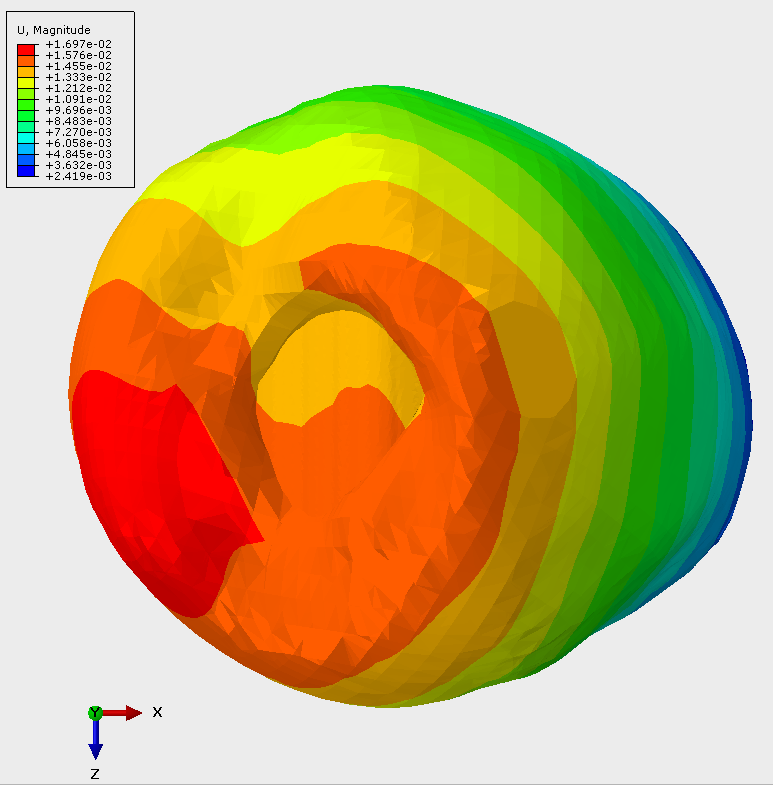


**Figure S39: Displacement Distribution Occlusal view of BM**


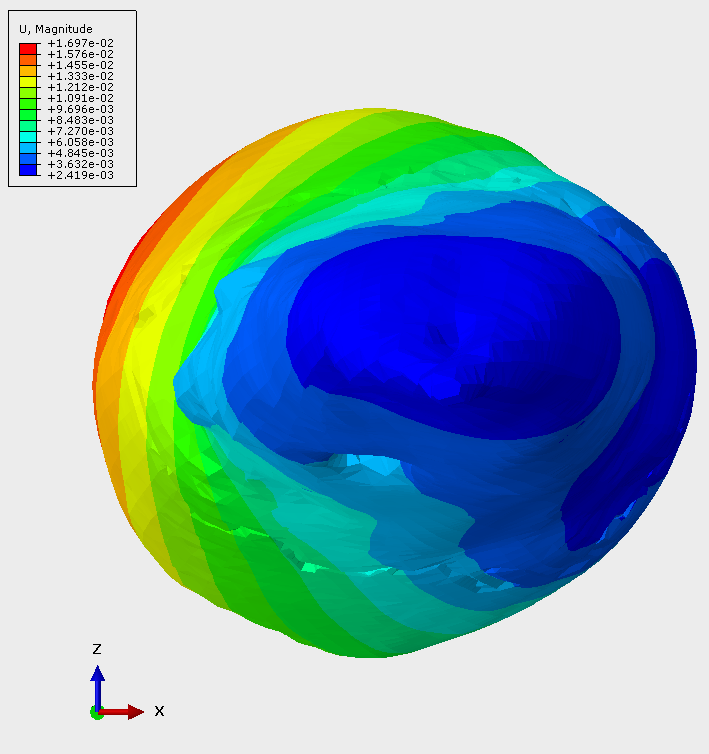


**Figure S40: Displacement Distribution Apical view of BM**


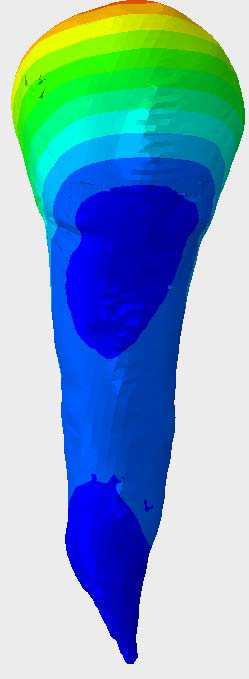

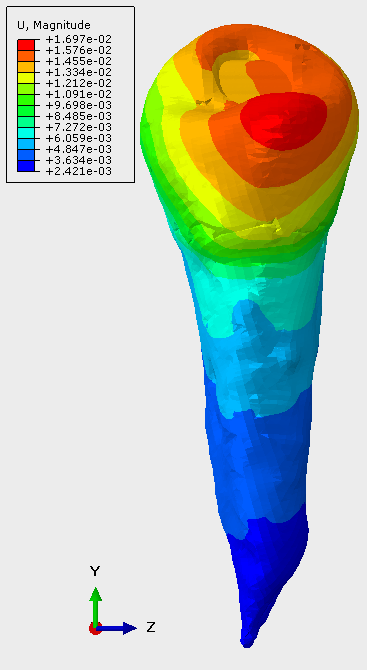


**Figure S41: Displacement Distribution Lingual/Buccal view of BM**

**Perforated & Repaired by Portland cement (PCM) (S42-S55)**


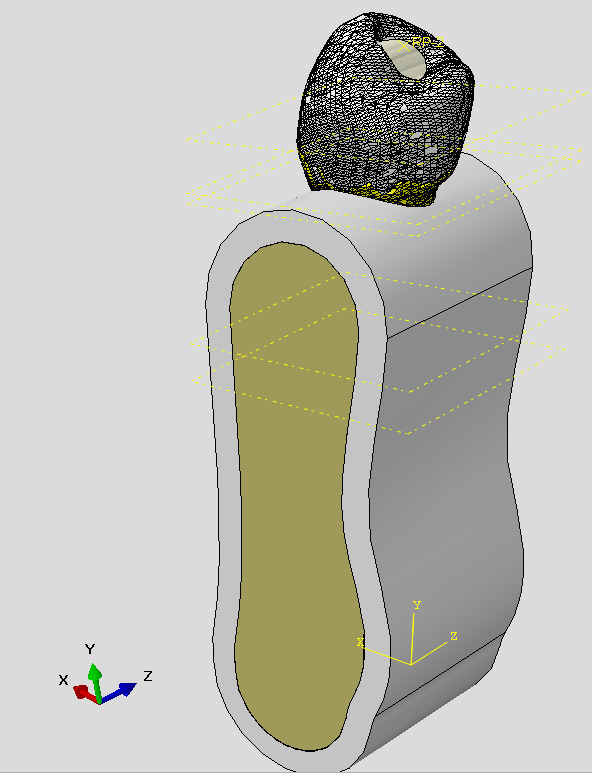


**Figure S42: Assembly 3D model of PCM**


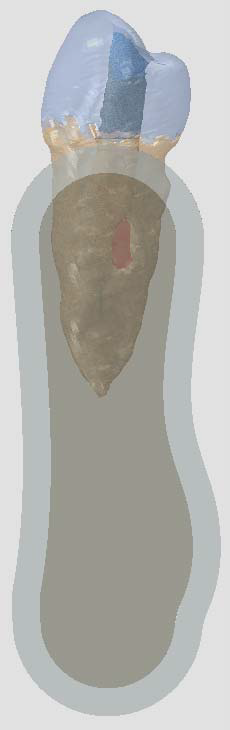

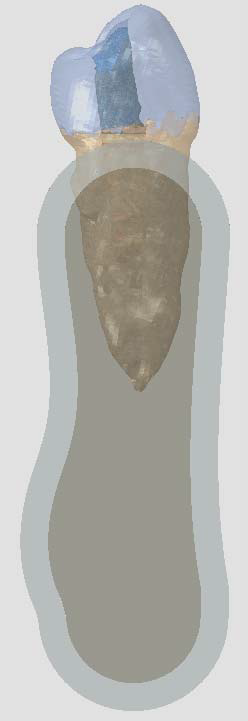


**(a) (b)**

**Figure S43: Assembly Section View (a) Distal, (b) Mesial of PCM**


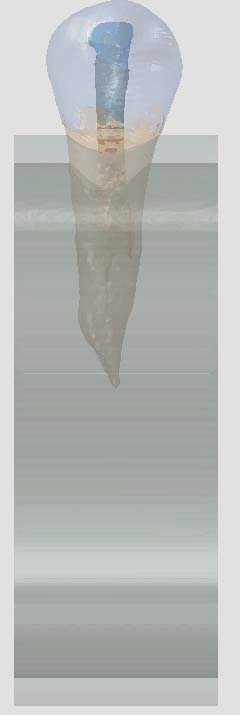

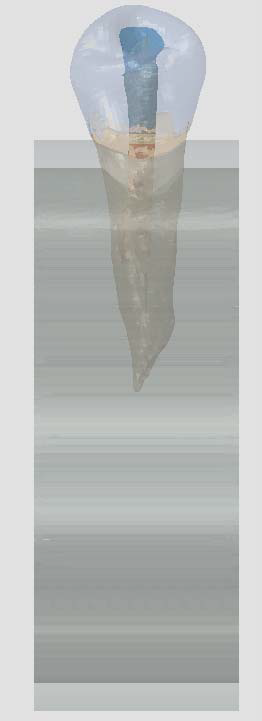


**(a) (b)**

**Figure S44: Assembly Section View (a) lingual, (b) Buccal of PCM**

**Mesh**


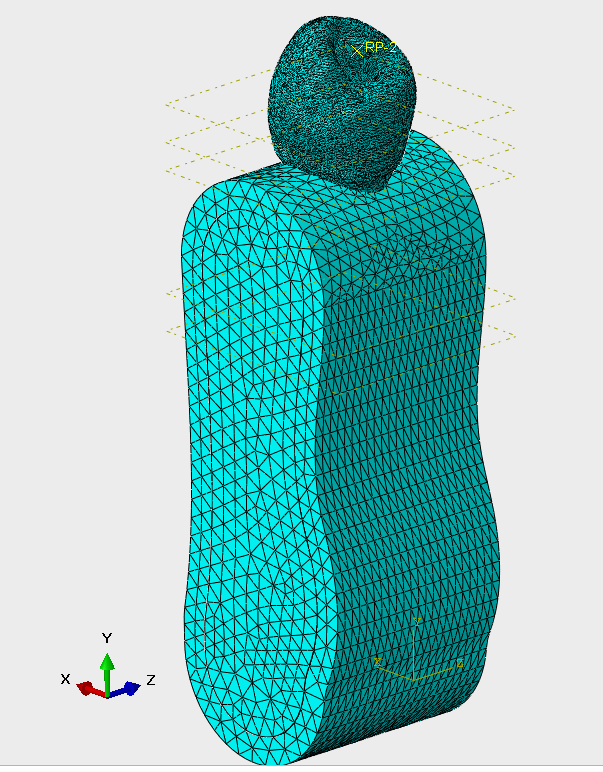


**Figure S45: Assembly Meshed 3D View**  **of PCM**

**Results of Scenario PCM model
Von –Mises Stress Distribution**

**Figure S46:**
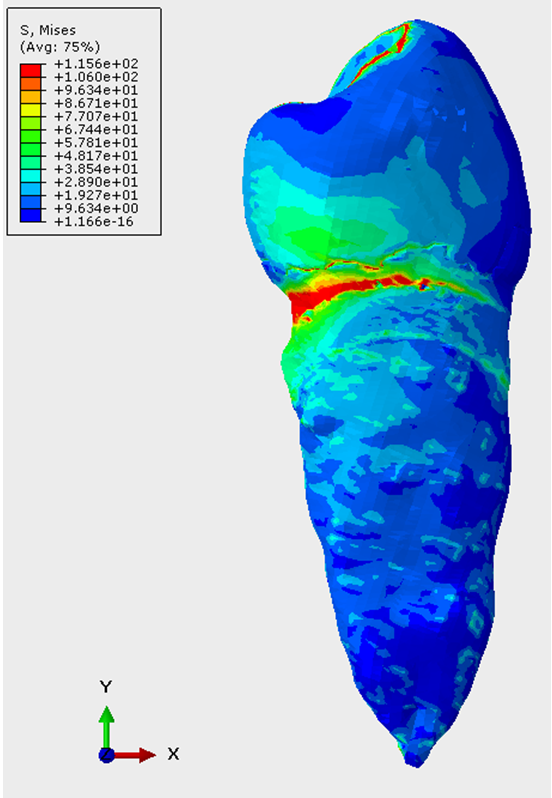
**Von –Mises Stress Distribution - Distal View of PCM**


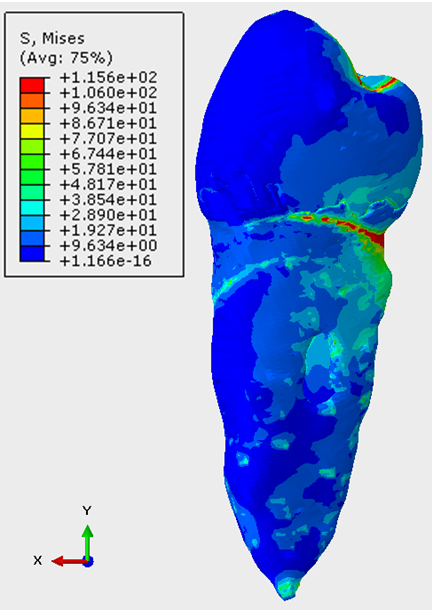


**Figure S47: Von –Mises Stress Distribution - Mesial View of PCM**


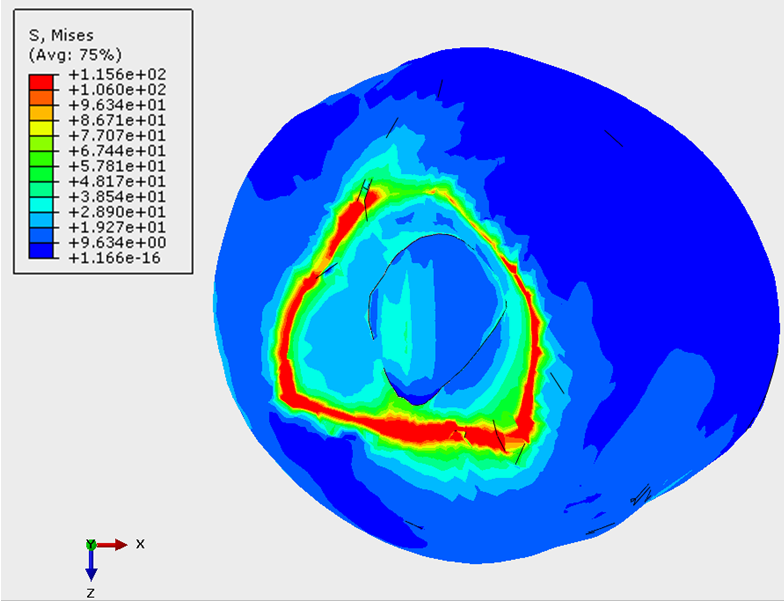


**Figure S48: Von –Mises Stress Distribution Occlusal View of PCM**


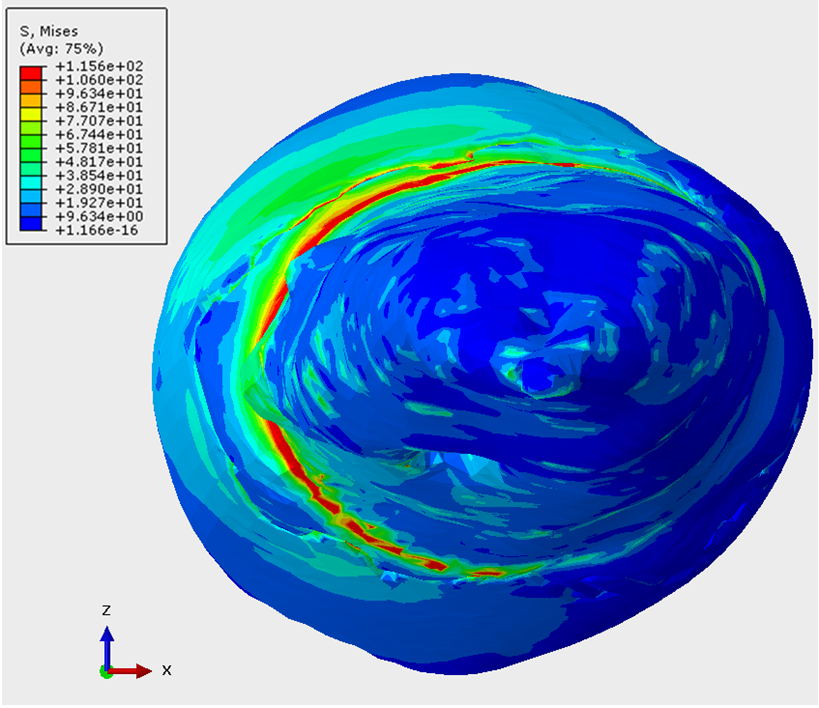


**Figure S49: Von –Mises Stress Distribution Apical View of PCM**


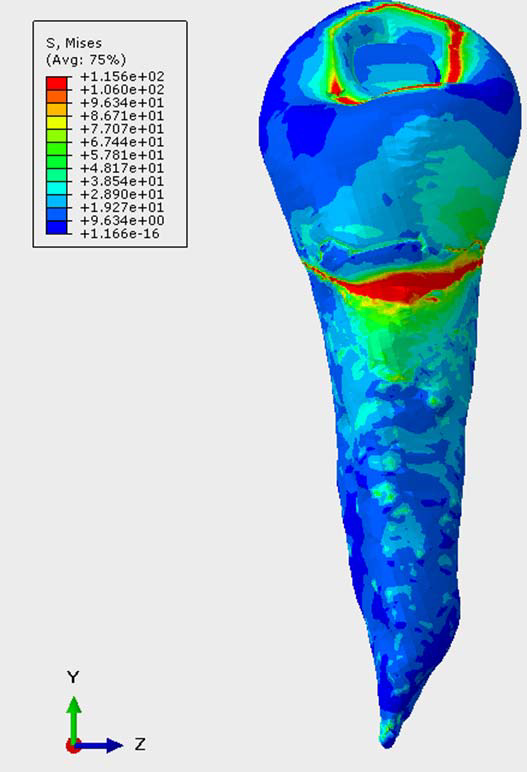


**Figure S50: Von –Mises Stress Distribution - Lingual View of PCM**


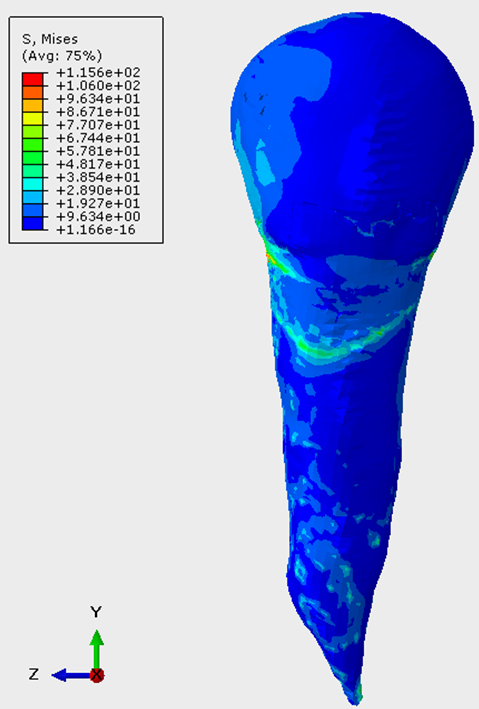


**Figure S51: Von –Mises Stress Distribution - Buccal View of PCM**

**Displacement Distribution Results**


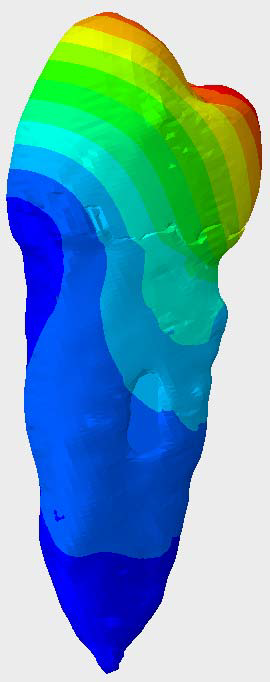

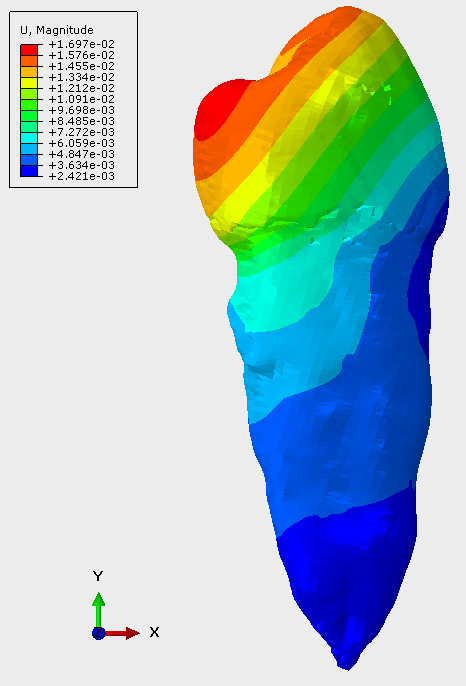


**Figure S52: Displacement Distribution Distal/Mesial view of PCM**


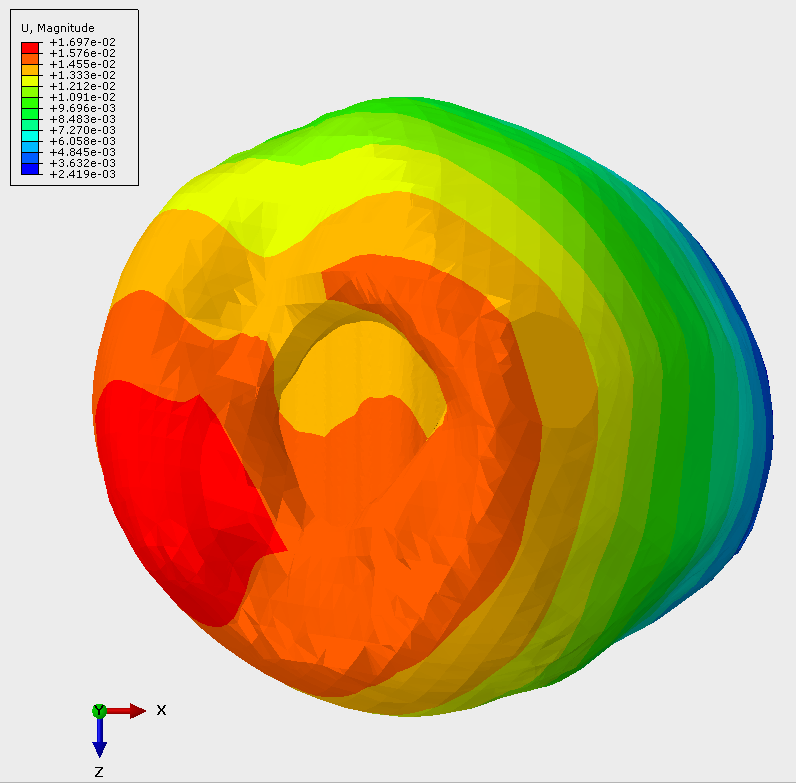


**Figure S53: Displacement Distribution Occlusal view of PCM**


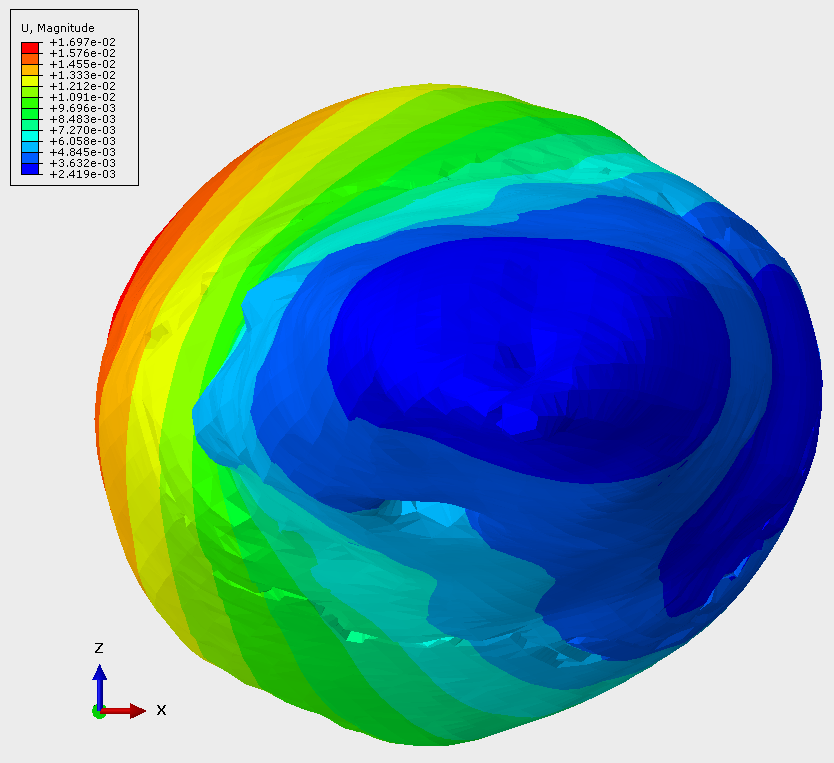


**Figure S54: Displacement Distribution Apical view of PCM**


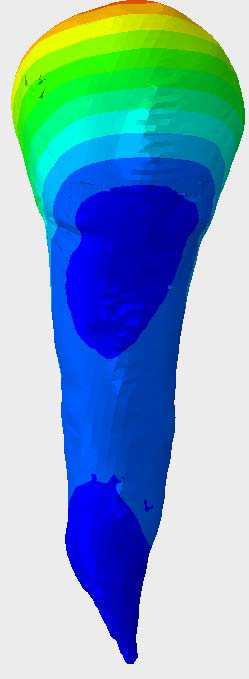

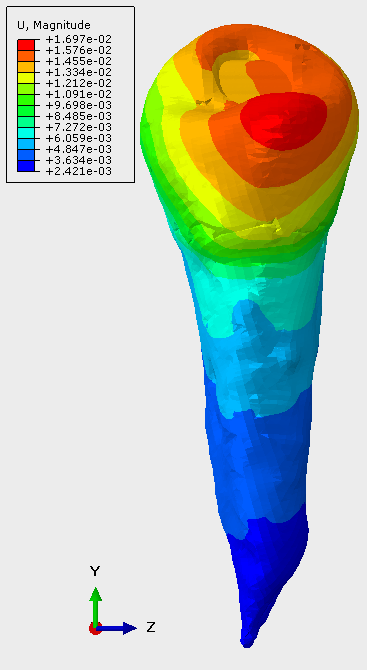


**Figure S55: Displacement Distribution Lingual/Buccal view of PCM**
